# Altered integrated and segregated states in cocaine use disorder

**DOI:** 10.3389/fnins.2025.1572463

**Published:** 2025-04-09

**Authors:** Yi Zheng, Yaqian Yang, Yi Zhen, Xin Wang, Longzhao Liu, Hongwei Zheng, Shaoting Tang

**Affiliations:** ^1^School of Mathematical Sciences, Beihang University, Beijing, China; ^2^Key Laboratory of Mathematics, Informatics and Behavioral Semantics, Beihang University, Beijing, China; ^3^Institute of Artificial Intelligence, Beihang University, Beijing, China; ^4^Zhongguancun Laboratory, Beijing, China; ^5^Beijing Advanced Innovation Center for Future Blockchain and Privacy Computing, Beihang University, Beijing, China; ^6^State Key Laboratory of Complex & Critical Software Environment, Beihang University, Beijing, China; ^7^Beijing Academy of Blockchain and Edge Computing, Beijing, China; ^8^Hangzhou International Innovation Institute, Beihang University, Hangzhou, China; ^9^Institute of Medical Artificial Intelligence, Binzhou Medical University, Yantai, China

**Keywords:** cocaine use disorder, resting-state functional MRI, integration–segregation, dynamic functional connectivity, graph theory

## Abstract

**Introduction:**

Cocaine use disorder (CUD) is a chronic brain condition that severely impairs cognitive function and behavioral control. The neural mechanisms underlying CUD, particularly its impact on brain integration–segregation dynamics, remain unclear.

**Methods:**

In this study, we integrate dynamic functional connectivity and graph theory to compare the brain state properties of healthy controls and CUD patients.

**Results:**

We find that CUD influences both integrated and segregated states, leading to distinct alterations in connectivity patterns and network properties. CUD disrupts connectivity involving the default mode network, frontoparietal network, and subcortical structures. In addition, integrated states show distinct sensorimotor connectivity alterations, while segregated states exhibit significant alterations in frontoparietal–subcortical connectivity. Regional connectivity alterations among both states are significantly associated with MOR and H3 receptor distributions, with integrated states showing more receptor-connectivity couplings. Furthermore, CUD alters the positive-negative correlation balance, increases functional complexity at threshold 0, and reduces mean betweenness centrality and modularity in the critical subnetworks. Segregated states in CUD exhibit lower normalized clustering coefficients and functional complexity at a threshold of 0.3. We also identify network properties in integrated states that are reliably correlated with cocaine consumption patterns.

**Discussion:**

Our findings reveal temporal effects of CUD on brain integration and segregation, providing novel insights into the dynamic neural mechanisms underlying cocaine addiction.

## 1 Introduction

Cocaine use disorder (CUD) is a chronic brain disorder characterized by compulsive cocaine use despite adverse consequences, difficulty in withdrawal, and intense cravings. With a worldwide prevalence of cocaine consumption rate surpassing 0.4% (World Drug Report, 2023; Morelos-Santana et al., 2024), CUD has emerged as a major public health concern. In the United States, it is reported that over 1.4 million individuals aged 12 years and above experienced a CUD in 2021 (Morelos-Santana et al., 2024). CUD has widespread effects on individuals, including reduced reward sensitivity (Volkow et al., 2010), impaired emotional regulation (Koob, 2008), and memory deficits (Hyman, 2005). Moreover, patients with CUD face an increased risk of developing mental and behavioral disorders (Oliva et al., 2021; Suchting et al., 2019). These severe cognitive and behavioral impairments highlight the necessity to elucidate the underlying neurobiological mechanisms of CUD, which will facilitate the development of novel therapeutic treatments.

Previous neuroimaging studies, primarily based on static functional networks derived from the total scan duration, have provided insights into the neural mechanisms of CUD to some extent. For instance, increased functional connectivity between the ventral caudate and the subgenual anterior cingulate cortex has been observed in CUD patients, indicating heightened impulsivity and higher relapse rates (Contreras-Rodríguez et al., 2015). Altered connectivity between reward networks and executive control networks has been associated with deficits in cognitive control (Hobkirk et al., 2019). Default mode network (DMN) related functional connectivity abnormalities have also been widely reported in addiction, impacting self-awareness and the regulation of negative emotions (Zhang and Volkow, 2019). Moreover, functional connectivity profiles in CUD have been found to correlate with the spatial densities of dopamine and serotonin receptors (Ricard et al., 2024). However, conventional static connectivity provides only an average representation of spatiotemporal patterns, neglecting the dynamic nature of neural processes. In contrast, dynamic functional connectivity (DFC) offers a more comprehensive examination of brain states by tracking changes in functional connectivity over time (Hutchison et al., 2013). One of the most widely used methods for investigating DFC is the sliding window approach (Allen et al., 2014), which divides functional magnetic resonance imaging (fMRI) time series into temporal windows, computes interregional correlations in each window, and generates a time-resolved sequence of connectivity matrices. A recent study using group ICA and a sliding window approach successfully identified distinct brain states in healthy controls and CUD patients, revealing state-dependent and state-shared hyperconnectivity patterns as well as differences in dynamic state proportions and duration (Cong et al., 2024).

Despite current progress, our understanding of brain dynamics underlying CUD remains incomplete. Specifically, the formal characterization of integration–segregation dynamics across time in CUD is still lacking. These two core attributes of brain function, commonly quantified using graph theory analysis, reflect distinct organizational principles (Sporns, 2013): integration supports efficient global communication across the whole network, while segregation facilitates specialized information processing within modules. For example, segregation is more prominent in simple motor tasks that involve independent processing of subsystems, whereas integration appears to dominate in cognitively challenging tasks that require coordination among multiple subsystems (Cohen and D’Esposito, 2016). Various addictive disorders have been shown to impact integration–segregation processes in the brain based on static functional networks. Alcohol use disorder reduced segregation within sensorimotor and association networks while adversely affecting network integration and striatal efficiency (Wang et al., 2024; Sjoerds et al., 2017). Male patients with Internet gaming disorder exhibited reduced modularity segregation (Zeng et al., 2021). Analyses of white matter functional networks in nicotine addiction demonstrated reduced segregation but relatively intact integration (Fan et al., 2023). In particular, analyses of static networks in CUD have provided substantial evidence of the altered integration and segregation. Connectome analyses revealed increased global connection strength and reduced global efficiency (the capacity for information transfer across the entire network, which reflects integration), local efficiency (the effectiveness of information processing within local subnetworks, which reflects segregation), and small-worldness (which quantifies the balance between local clustering and global integration, reflecting the integration–segregation tradeoff) in CUD cohorts (Wang et al., 2015). Differences in network efficiency were also observed between early and long-term abstinence in cocaine addiction (Zilverstand et al., 2023). Module-level analyses revealed altered connectivity between the salience network and DMN (Liang et al., 2015), indicating a disrupted segregation function. Moreover, at the neurochemical level, CUD is characterized by dopaminergic hypofunction, which is suggested to be associated with increased global integration, decreased segregation, and enhanced connections between the normally negatively correlated task-positive and default-mode networks (Carbonell et al., 2014; Shine et al., 2019). These abnormalities in static network structure or neurotransmitter systems likely reflect or elicit integration–segregation deficits in a finer-grained temporal scale of cocaine addiction, yet empirical research remains lacking.

In this study, we employed a graph theory-based framework to explore integration, segregation, and their dynamic instantiation (Shine et al., 2016). This method has been widely applied to extract reliable representations of predominantly integrated and segregated states from DFC, as well as the temporal transitions between them. These states have been shown to reflect cognitive and motor functions (Shine et al., 2016), exhibit different couplings with underlying structural connectivity (Fukushima et al., 2018), and be influenced by levels of consciousness (Luppi et al., 2019, 2021b) and psychedelics (Luppi et al., 2021a). Here, we aimed to explore the time-resolved effects of CUD on the brain’s integration–segregation states. We first examined differences in state time series between healthy controls and CUD. Subsequently, we evaluated the alterations of dynamic functional connectivity induced by CUD in each state and explored possible correlations with spatial receptor and transporter densities. We also employed graph theory analysis to reveal aberrant network organization. Our focus was not only on the shared characteristics of integrated and segregated states but also on their differences to understand the temporal effects of CUD on brain function comprehensively. Finally, we correlated network properties with cocaine consumption patterns to show their potential for tracking disease trajectories.

## 2 Materials and methods

### 2.1 Methods overview

This study investigated the impact of cocaine use disorder (CUD) on brain dynamic integration and segregation. We analyzed resting-state fMRI data from the publicly available SUDMEX-CONN dataset (National Institute of Psychiatry, Mexico City, Mexico) (Angeles-Valdez et al., 2022) to test whether CUD would disrupt integrated and segregated functional states, leading to altered connectivity patterns and network topology. First, we preprocessed the functional images using fMRIPrep (Poldrack Lab, Stanford, CA, United States) (Esteban et al., 2019) and denoised them with XCP-D (Lifespan Informatics and Neuroimaging Center, Philadelphia, PA, United States) (Mehta et al., 2024), followed by parcellation using brain atlases. Second, we analyzed dynamic functional connectivity using the sliding window approach (Allen et al., 2014) and cartographic profiling (Shine et al., 2016) to extract representative integrated and segregated states. Third, we employed network-based statistic (NBS) to analyze connectivity differences (Zalesky et al., 2010) and explored potential associations with receptors and transporters. Finally, we examined differences in network properties using graph theory analysis and investigated their possible associations with cocaine consumption patterns.

### 2.2 Participants

This study utilized neuroimaging data from the publicly available SUDMEX-CONN dataset (Angeles-Valdez et al., 2022). All procedures adhered to the Declaration of Helsinki and were approved by the Ethics Committee of the Instituto Nacional de Psiquiatría. All participants, including healthy controls (HCT) and patients with CUD, provided oral or written informed consent. The inclusion criteria of this study for CUD patients are the following: (1) Active cocaine use or abstinence duration less than 60 days prior to the scan; (2) minimum cocaine use frequency of 3 times weekly; and (3) no sustained abstinence periods exceeding 60 consecutive days within the last 12 months. The participants were required to abstain from any drug use prior to and on the day of the study. After excluding subjects with excessive head motion (mean framewise displacement >0.55 mm) (Ricard et al., 2024; Parkes et al., 2018), the final cohort comprised 130 subjects (62 HCT and 68 CUD, 83% male, mean age = 30.90 ± 7.69, age range = 18–50, years since the beginning of cocaine use: 10.24 ± 6.81, and cocaine age of onset: 21.13 ± 5.80).

### 2.3 MRI data acquisition

The structural and functional data were acquired using a Philips Ingenia 3 T MR system with a 32-Channel dS Head Coil (Philips Healthcare, Eindhoven, Netherlands). Briefly, the resting state fMRI data was acquired using a gradient recalled echo planar imaging sequence (dummies = 5, TR = 2,000 ms, TE = 30.001 ms, 3.0-mm isotropic voxels, flip angle = 75°, field of view [FOV] = 240 mm × 240 mm, slice acquisition order = interleaved (ascending), number of slices = 36, and phase encoding direction = AP). The participants viewed a fixation cross during the 10-min scan. T1w images were collected using a three-dimensional Fast Field Echo with SENSitivity Encoding (FFE SENSE) sequence (repetition time [TR] = 7 ms, echo time [TE] = 3.5 ms, FOV = 240 mm × 240 mm, number of slices = 180, gap = 0, plane = sagittal, and 1.0-mm isotropic voxels [the first five participants were acquired with a voxel size = 0.75 mm × 0.75 mm × 1 mm]).

### 2.4 Preprocessing and time series generation

The fMRIPrep was utilized to perform data preprocessing (Esteban et al., 2019; Esteban et al., 2020). The TI-weighted (T1w) image preprocessing included intensity non-uniformity correction, skull stripping, tissue segmentation (cerebrospinal fluid, white matter, and gray matter), brain surface reconstruction, nonlinear registration, and normalization to standard volumetric spaces. Functional scans underwent head motion correction and slice-time correction and were co-registered to the T1w reference. Confound time series, including framewise displacement (FD) and component-based method (CompCor), were calculated.

The XCP-D was employed to denoise the preprocessed fMRI data with the “acompcor_gsr” strategy (Mehta et al., 2024). This process included discarding the first five non-steady volumes, removing the top 5 aCompCor components, and 6 motion parameters with their temporal derivatives. Spatial smoothing was applied using a Gaussian kernel (Full Width at Half Maximum [FWHM] = 8 mm). Following previous studies (Ricard et al., 2024; Chan et al., 2021), we implemented global signal regression, as evidence indicated that a substantial portion of the global signal reflects spatially non-specific noise induced by head motion (Power et al., 2014; Power et al., 2015; Power et al., 2017; Satterthwaite et al., 2013). Explicitly removing the global signal effectively minimizes these known artifacts (Power et al., 2014; Satterthwaite et al., 2013; Yan et al., 2013).

To extract regional time series, we parcellated the cleaned data using a 232-node atlas (named Schaefer-232), which combines the Schaefer-200 cerebral atlas (Computational Brain Imaging Group, Singapore) (Schaefer et al., 2018) with the Melbourne subcortical atlas (scale-2) (systems lab, Melbourne, VIC, Australia) (Tian et al., 2020). This widely adopted atlas produces network topologies that are highly representative among available parcellation schemes (Luppi and Stamatakis, 2021). We applied band-pass filtering (0.01–0.08 Hz) to focus on the frequency range most relevant to resting-state functionality (Deco et al., 2017; Deco et al., 2021). For validation purposes, we provided results using a different Brainnetome atlas (Brainnetome Center, Beijing, China) (Fan et al., 2016) in Supplementary material.

### 2.5 Dynamic functional connectivity analysis

#### 2.5.1 Sliding window approach

**Figure 1:**
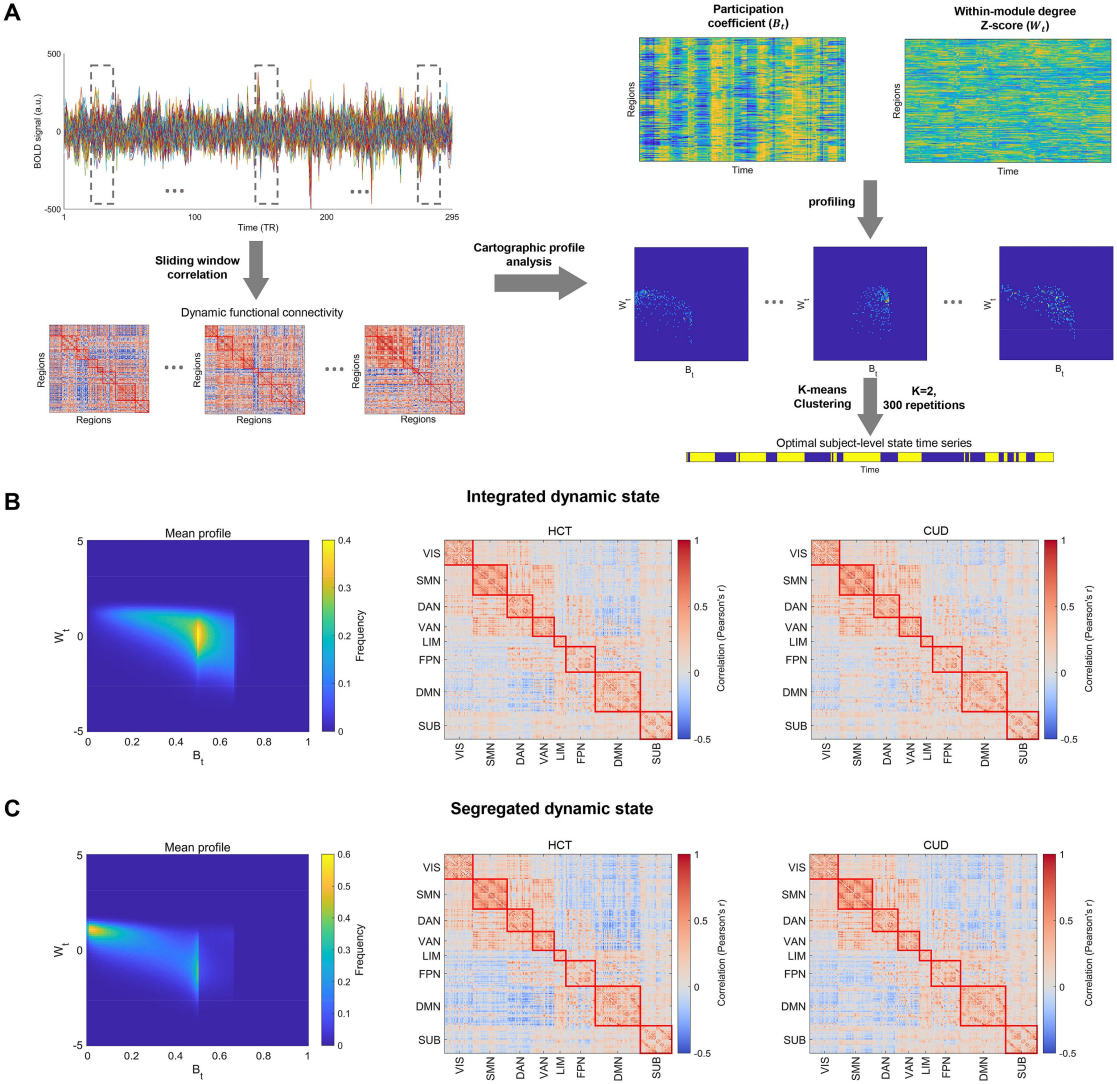
Integrated and segregated dynamic states identified using cartographic profile analysis. **(A)** Dynamic functional connectivity is computed using sliding window correlations. At each time window, participation coefficients and within-module degree Z-scores are calculated for each region and aggregated into a joint histogram as the cartographic profile. The k-means clustering (k = 2, 300 repetitions) was applied to the cartographic profiles of each subject, generating optimal subject-level state time series. **(B)** The mean profile and centroids in HCT and CUD groups for the integrated state. **(C)** The mean profile and centroids in HCT and CUD groups for the segregated state. Brain regions are organized by Yeo 7 functional networks and subcortical structures (VIS, visual network; SMN, sensorimotor network; DAN, dorsal attention network; VAN, ventral attention network; FPN, frontoparietal network; DMN, default mode network; SUB, subcortical structures).

We employed an overlapping sliding window approach to construct dynamic functional connectivity (Allen et al., 2014) (Figure 1A). A 44-s (22 TR) window with 1-TR steps was used, aligning with previous studies (Luppi et al., 2019, 2021a). Within each window, Pearson correlations between brain regions were calculated to generate dynamic connectivity matrices.

#### 2.5.2 Cartographic profiling

The cartographic profile method was used to characterize the integration and segregation of dynamic networks (Shine et al., 2016; Fukushima et al., 2018; Luppi et al., 2019, 2021a; Zarkali et al., 2022). At each time t we applied the Louvain algorithm to identify community structures, using an asymmetric version for signed networks (Rubinov and Sporns, 2011). The community label of each node is iteratively adjusted to maximize the modularity function, $Q_{t}$ :


$$Q_{t}=\frac{1}{v^{+}}\sum_{ij}\left(w_{ij}^{+}-e_{ij}^{+}\right)\delta_{M_{i}M_{j}}-\frac{1}{v^{+}+v^{-}}\sum_{ij}\left(w_{ij}^{-}-e_{ij}^{-}\right)\delta_{M_{i}M_{j\text{.}}}$$

where v represents the total functional weight, $w_{ij}$ denotes the signed connection weight between the regions i and j , $e_{ij}$ is the corresponding chance-expected connection weight, and $\delta_{M_{i}M_{j}}$ equals 1 if the regions i and j are in the same module and 0, otherwise; the symbols “+” and “–” represent the positive and negative edges, respectively.

The maximum $Q_{t}$ value represents the optimal modular structure at the time t , characterized by strong positive within-module connections and weak or negative between-module connections (Sporns and Betzel, 2016). We generated a consensus partition by repeating this process 100 times with the resolution parameter γ = 1.

Next, based on the identified network modules, we calculated the participation coefficient and within-module degree Z-score for each region. The participation coefficient was defined as


$$B_{\mathrm{it}}=1-\sum_{s=1}^{n_{M}}{\left(\frac{K_{ist}}{K_{\mathrm{it}}}\right)}^{2}$$

where $K_{\mathrm{it}}$ represents the total positive weight of the region i at the time t , $K_{ist}$ represents the positive weight between the region i and the other regions in the module s at the time t , and $n_{M}$ is the number of modules in the network. The value of the participation coefficient ranges from 0 to 1, quantifying the dispersion of regional connections across modules. Higher participation coefficients suggest that connections are distributed across multiple modules, supporting intermodular information integration. The within-module degree Z-score was computed as


$$W_{\mathrm{it}}=\frac{K_{ist}-{\bar{K}}_{ist}}{\sigma_{K_{ist}}}$$

where $K_{ist}$ represents the total connection weight between the region i and other regions in the module s at the time t , and ${\bar{K}}_{ist}$ and $\sigma_{K_{ist}}$ are the mean and standard deviation of K across all nodes in the module s . A higher within-module degree Z-score indicates a crucial role in internal information flow.

The participation coefficients and within-module degree Z-scores of all nodes were aggregated into a joint histogram, representing the “cartographic profile” at the time t (Shine et al., 2016) (Figure 1A). Each subject had 295 cartographic profiles.

#### 2.5.3 Integrated and segregated states

We applied the k-means clustering algorithm to classify each subject’s cartographic profiles into two clusters, which correspondingly separated the dynamic functional connectivity (Shine et al., 2016; Luppi et al., 2019, 2021a) (Figure 1A). We used the Pearson correlation as the distance metric, repeated clustering 300 times with random initializations, and selected the optimal separation as the final solution. Clusters with higher mean participation coefficients were identified as “integrated” states, while those with lower coefficients were labeled as “segregated” states (Shine et al., 2016). In this study, we focused on cluster number k = 2, as it reflects two extremes of topological properties. Using the Silhouette score, we also evaluated clustering quality for k = 2–7. We calculated each subject’s average dynamic functional connectivity in each state as state-specific centroids. We organized brain regions according to the Yeo 7 functional networks (Computational Brain Imaging Group, Singapore) and subcortical structures. The functional networks included the visual network (VIS, involved in processing visual information and spatial perception); the sensorimotor network (SMN, responsible for motor control and somatosensory processing); the dorsal attention network (DAN, associated with goal-directed attention and visuospatial processing); the ventral attention network (VAN, related to stimulus-driven attention and the detection of salient stimuli); the frontoparietal network (FPN, implicated in cognitive control and executive functions); and the default mode network (DMN, engaged in self-referential thinking and mind-wandering). Additionally, subcortical structures (SUB) were included to account for their role in regulating autonomic functions and reward processing. These abbreviations were used throughout the text and figures to facilitate the interpretation of network analysis results.

#### 2.5.4 Dynamic measures

To characterize temporal dynamics of brain states, we computed five measures from the state time series obtained through k-means clustering: (1) fraction time (the proportion of time spent in each state), (2) dwell time (the average duration of continuous periods in each state), (3) total transitions (the number of transitions between states), (4) sample entropy (the regularity of state patterns), and (5) Lempel–Ziv complexity (pattern diversity in state sequences).

### 2.6 Graph theory analysis

We employed multiple global graph metrics from the Brain Connectivity Toolbox (Computational Cognitive Neuroscience Laboratory, Bloomington, IN, United States) (Rubinov and Sporns, 2010) to systematically examine network-level differences between HCT and CUD conditions. Our analysis focused on three aspects of brain network properties: strength and efficiency metrics, small-world properties, and community structure. Following prior recommendations (Van Den Heuvel et al., 2017), we used weighted matrices to preserve the continuous nature of functional connections. This analysis was applied to both integrated and segregated state centroids, as well as the corresponding subnetworks identified through NBS (Morand-Beaulieu et al., 2023; Myung et al., 2016; Román et al., 2017) (see Section 2). Note that it is meaningful to evaluate segregation metrics during integrated states or integration metrics during segregated states, as these properties are neither dichotomous nor mutually exclusive (Luppi et al., 2021a), allowing for a nuanced characterization of dynamic brain states.

Specifically, network measures related to strength and efficiency include positive strength ( $S_{pos}$ , mean node-level positive strength), negative strength ( $S_{neg}$ , mean node-level negative strength), global efficiency ( $E_{\mathrm{global}}$ , average inverse shortest path length in the whole network), local efficiency ( $E_{\mathrm{local}}$ , average inverse shortest path length in the neighboring network), and functional complexity at different thresholds (Zamora-López et al., 2016) (functional complexity reflects the similarity between the empirical connectivity distribution and a uniform distribution). Small-world properties include the clustering coefficient ( C , the mean tightness of connections between nodes and their neighbors), characteristic path length ( L , average shortest path length between nodes in the network), normalized clustering coefficient ( nC ), normalized characteristic path length ( nL ), and small-worldness (SW, the ratio of the normalized clustering coefficient and normalized characteristic path length). The measures associated with community structure include mean betweenness centrality (Girvan and Newman, 2002) ( mBC , the average role of nodes as intermediaries in the shortest paths) and modularity ( Q , a measure of the quality of community partitioning). A detailed information on these graph theory measures is provided in Supplementary information.

### 2.7 Statistical analysis

Network-based statistic (NBS) was employed to examine cocaine-induced differences in dynamic functional connectivity across integrated and segregated brain states (Zalesky et al., 2010). NBS is well-suited for network data, providing greater statistical power than multiunivariate analyses. This approach begins by performing single statistical tests for each connection to create a statistical matrix. Subsequently, it applies thresholding to identify significant connections and forms connected components, whose statistical significance is evaluated against null models generated through random permutations. We conducted an analysis of covariance (ANCOVA) for edgewise statistical tests, controlling for age, sex, education, and mean framewise displacement, and chose the component forming threshold as 0.05. Robust analyses using the Brainnetome atlas and a stricter threshold (0.01) are provided in Supplementary material.

Inspired by previous research linking static connectivity patterns of CUD to neurotransmitter receptor densities (Ricard et al., 2024), we investigated associations between dynamic functional connectivity alterations and neurotransmitter expression in integrated and segregated states. Using Neuromaps (Markello et al., 2022), we extracted receptor and transporter data for nine neurotransmitters and parcellated them using the same atlas to derive their topological distribution. We computed Spearman correlations between regional receptor and transporter densities and the number of significantly altered connections identified through NBS, as Spearman correlation is appropriate given the abnormally high values of these two measures observed in specific brain regions (Ricard et al., 2024). The statistical significance was determined using permutation tests (10,000 permutations): spin-tests for cortical regions with spatial autocorrelation preserved (Váša et al., 2018), and random shuffling for subcortical regions within each hemisphere (Alexander-Bloch et al., 2018; Markello and Misic, 2021). The *p*-values were false discovery rate (FDR)-corrected for multiple receptors and transporters. For receptors and transporters with multiple density maps,we performed additional correlation analyses as reproducibility tests. To further assess the robustness of our findings, we performed correlation analyses based on the Brainnetome atlas. The main text focused on significant correlations across discovery analysis, reproducibility tests (when multiple maps were available), and cross-atlas validation.

The graph theory metrics were compared between HCT and CUD groups using ANCOVA, adjusting for age, sex, education, and mean framewise displacement. FDR correction was applied for integrated and segregated states. We then conducted Pearson’s partial correlation analyses to investigate potential relationships between network metrics and cocaine consumption patterns (years since the beginning of cocaine use and cocaine age of onset). We controlled age, sex, education, and mean framewise displacement as covariates and set the significance level at *p* < 0.05. Given the exploratory nature of the analysis, no multiple comparison corrections were applied (Luppi et al., 2021a). To minimize false positives, only significant results across both atlases were reported in the main text, indicating robustness across different atlas types.

## 3 Results

### 3.1 Preserved integration–segregation dynamics under CUD

We employed cartographic profile analysis to classify dynamic functional connectivity into integrated and segregated states. This approach enabled us to investigate the underlying brain dynamics in cocaine use disorder (CUD).

We validated the effectiveness of this method by showing clustering performance. As shown in Figures 1B,C, the approach successfully identified clusters with distinct topological properties, despite subtle differences in their centroids. The mean cartographic profiles aligned well with previous findings (Shine et al., 2016; Luppi et al., 2021b), with integrated states showing significantly higher mean participation coefficients than segregated states in both healthy controls (HCT) and CUD groups (Supplementary Figure 1). The Brainnetome atlas analysis yielded similar results, confirming the robustness (Supplementary Figure 2). Moreover, cluster quality assessment using Silhouette scores (k = 2–7) revealed optimal clustering at k = 2 (Supplementary Figure 3), supporting the integrated-segregated state classification. The fraction of time spent in the integrated state for HCT (mean = 0.64, standard deviation [SD] = 0.11) was also consistent with previous studies (Shine et al., 2016; Luppi et al., 2021a, 2021b).

The analysis of temporal dynamics, including fraction time, dwell time, total transitions, sample entropy, and Lempel–Ziv complexity, showed no significant differences between CUD and HCT (Supplementary Table 1), suggesting preserved temporal state dynamics in CUD.

### 3.2 Temporal effects of CUD on brain connectivity

**Figure 2:**
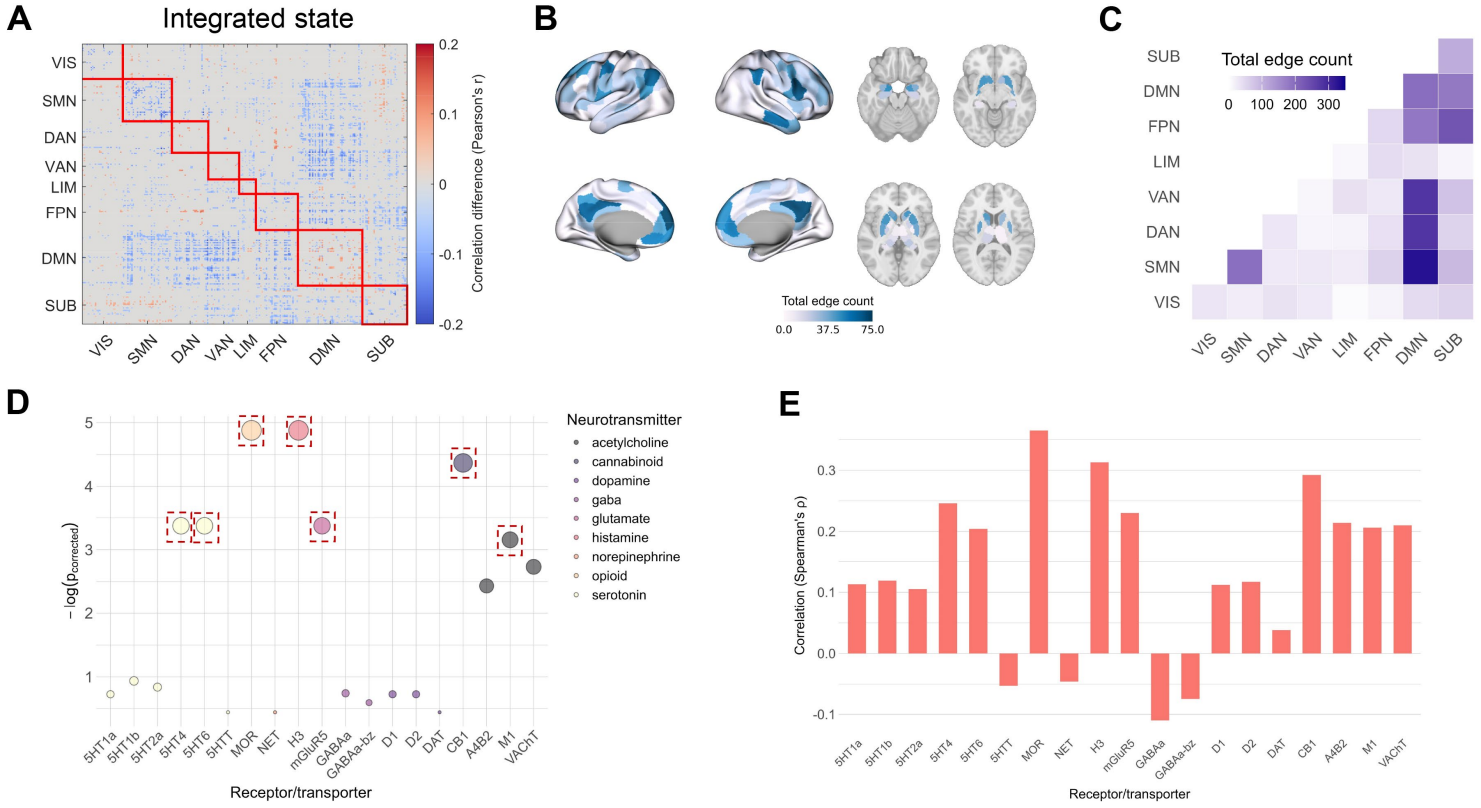
Cocaine-induced changes in dynamic functional connectivity of the integrated state. **(A)** Network-based statistic (NBS) analysis showing significant connectivity differences (red: HCT > CUD; blue: CUD > HCT). These values were obtained by taking the differences between the Fisher Z-transformed correlation coefficients and converting them back to correlation values. **(B)** The count of significant edges at each region rendered on the cortical surface and subcortical regions. **(C)** Network-level analysis showing total significant edge count within and between functional networks. **(D)** Significance of correlations between regional edge counts and neurotransmitter receptor/transporter densities (FDR-corrected *p*-values shown as negative logarithms). Red squares indicate *p* < 0.05 across discovery, replication (when applicable), and cross-atlas analyses. **(E)** Spearman’s correlation coefficients between regional edge counts and receptor/transporter densities.

**Figure 3:**
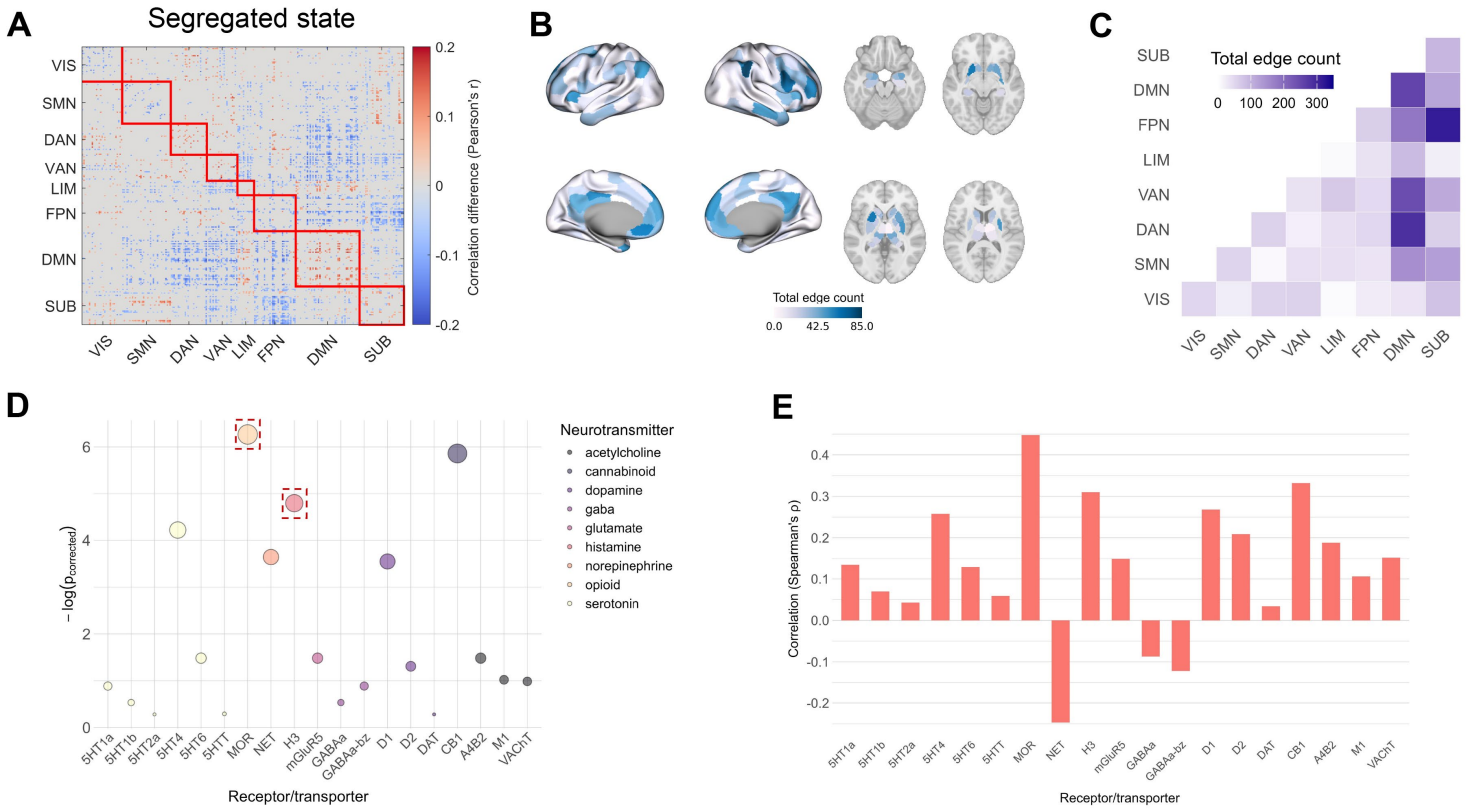
Cocaine-induced changes in dynamic functional connectivity of the segregated state. **(A)** NBS analysis showing significant connectivity differences (red: HCT > CUD; blue: CUD > HCT). **(B)** The count of significant edges at each region rendered on the cortical surface and subcortical regions. **(C)** Network-level analysis showing total significant edge count within and between functional networks. **(D)** Significance of correlations between regional edge counts and neurotransmitter receptor/transporter densities (the FDR-corrected *p*-values shown as negative logarithms). Red squares indicate *p* < 0.05 across discovery, replication (when applicable), and cross-atlas analyses. **(E)** Spearman’s correlation coefficients between regional edge counts and receptor/transporter densities.

Next, we explored the effects of cocaine on brain states by examining functional connectivity patterns. Using NBS, we identified significant network alterations between HCT and CUD for both integrated and segregated dynamic states (integrated state *p* = 0.046; segregated state *p* = 0.017) (Figures 2A, 3A). The majority of significant connectivity showed increased strength in the CUD condition. Given the widespread connectivity alterations, we quantified the total count of altered edges for each brain region and mapped these onto cortical and subcortical structures (Figures 2B, 3B). In the integrated state, the most pronounced changes occurred in the left prefrontal cortex, precuneus posterior cingulate cortex, and right precentral ventral region. The segregated state showed the highest edge counts in the right inferior parietal lobule, right precentral ventral region, prefrontal cortex, and left anterior putamen. Network-level analysis revealed that in the integrated state, significant connectivity alterations were most prominent between DMN-SMN, DMN-DAN, DMN-VAN, and SUB-FPN, as well as within DMN and SMN (Figure 2C). The segregated state showed major alterations between SUB-FPN, DMN-DAN, DMN-VAN, and within DMN (Figure 3C). These patterns were robust across the Brainnetome atlas and threshold 0.01 (Supplementary Figures 4, 5), except for weak DMN-VAN alterations in the segregated state using the Brainnetome atlas. While both dynamic states showed similar alteration patterns, notable differences emerged, especially within SMN and between SMN-DMN and SUB-FPN (Supplementary Figure 6).

We computed Spearman’s correlations between the spatial distribution of regional significant edge counts and neurotransmitter receptor and transporter densities (Figures 2D,E, 3D,E). In the integrated state, significant correlations (FDR-corrected) were found with receptor densities of 5HT4 (ρ = 0.25, *p* = 0.034), 5HT6 (ρ = 0.20, *p* = 0.034), MOR (ρ = 0.37, *p* = 0.008), H3 (ρ = 0.31, *p* = 0.008), mGluR5 (ρ = 0.23, *p* = 0.034), CB1 (ρ = 0.29, *p* = 0.013), and M1 (ρ = 0.21, *p* = 0.043). These associations remained robust when tested using the Brainnetome parcellation (Supplementary Table 2) and additional positron emission tomography (PET) maps (when available) (Supplementary Table 3). In the segregated state, edge patterns significantly correlated with receptor and transporter densities of 5HT4 (ρ = 0.26, *p* = 0.015), MOR (ρ = 0.45, *p* = 0.002), NET (ρ = −0.25, *p* = 0.026), H3 (ρ = 0.31, *p* = 0.008), D1 (ρ = 0.27, *p* = 0.029), and CB1 (ρ = 0.33, *p* = 0.003). However, only MOR and H3 receptor correlations remained consistent across the Brainnetome parcellation (Supplementary Table 4) and additional PET maps (when available) (Supplementary Table 5).

### 3.3 Network organization changes across different dynamic states

In addition to changes at the functional connectivity level, CUD may also disrupt the global topological organization of functional networks in both integrated and segregated states. To test this hypothesis, we compared three categories of topological properties between HCT and CUD conditions and examined whether these metrics showed variability across different states. Graph theory metrics were applied to both dynamic functional states and NBS-constrained subnetworks. While whole-network analysis revealed no significant differences between conditions (Supplementary Table 6), subnetwork analysis yielded several key findings. We also presented results of static subnetworks constrained by the corresponding NBS for comparison (Supplementary Table 7).

**Figure 4:**
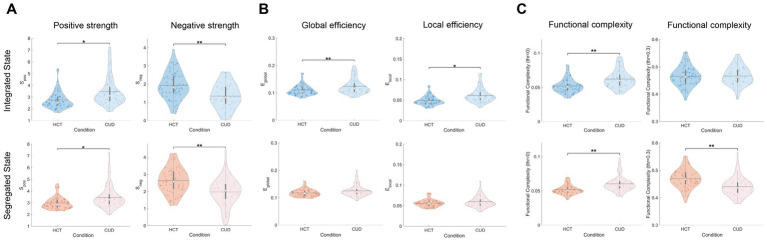
Strength and efficiency alterations in NBS subnetworks induced by cocaine. **(A)** Violin plots of positive and negative strength across different conditions and dynamic states. **(B)** Violin plots of global and local efficiency across conditions and states. **(C)** Violin plots of functional complexity with various thresholds across conditions and states. The colored dots represent individual data, box limits indicate the 25th and 75th percentiles, horizontal lines mark the medians, white dots represent the mean, and whiskers cover the 1.5 interquartile range. The *p*-values were FDR-corrected using the Benjamini–Hochberg procedure; *p < 0.05 and **p < 0.001.

First, we investigated subnetwork properties of strength and efficiency. CUD showed significantly higher positive strength (integrated: *F* = 9.84, *p* = 0.002; segregated: *F* = 7.76, *p* = 0.006) and lower negative strength (integrated: *F* = 13.97, *p* < 0.001; segregated: *F* = 13.09, *p* < 0.001) across both states (Figure 4A), suggesting altered balance between positive and negative correlation. Moreover, the global and local efficiency was higher in CUD compared to HCT during the integrated state (global: *F* = 15.71, *p* < 0.001; local: *F* = 5.54, *p* = 0.020) and no significant differences were found during the segregated state (Figure 4B). Analysis of functional complexity revealed threshold-dependent effects (Figure 4C). At threshold 0, CUD showed higher complexity across both states (integrated: *F* = 14.94, *p* < 0.001; segregated: *F* = 16.53, *p* < 0.001). However, at threshold 0.3, CUD exhibited significantly lower complexity only in the segregated state (*F* = 14.15, *p* < 0.001), indicating potential temporal preference. This result was robust across various thresholds (Supplementary Table 1). Moreover, no significant differences were observed in the complexity of static functional connectivity at the same high thresholds (Supplementary Table 7). The Brainnetome atlas analysis largely replicated our main findings (Supplementary Table 8), with the exception that global and local efficiency were significantly higher in CUD across both integrated and segregated states.

**Figure 5:**
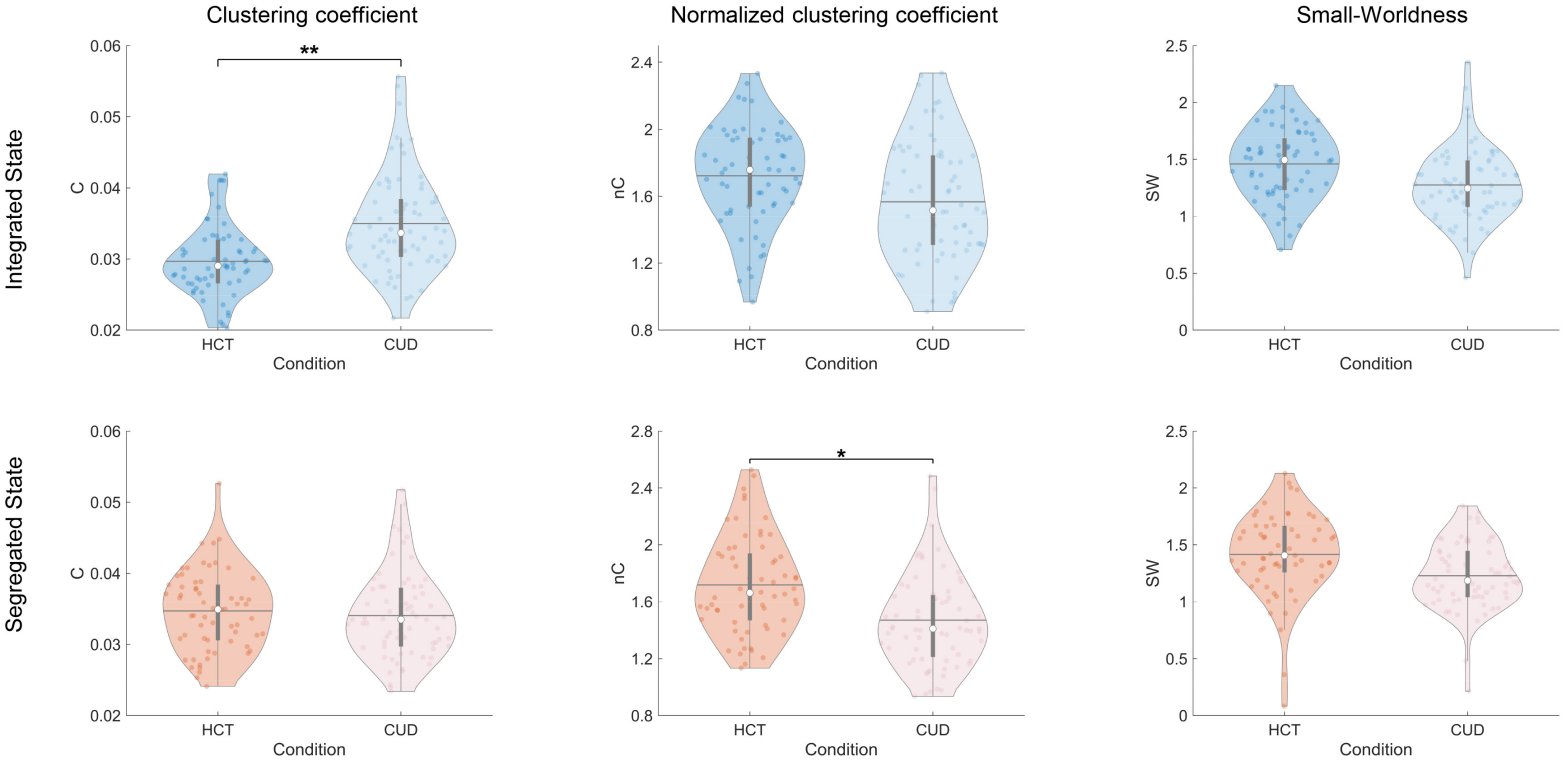
Aberrant clustering coefficients of NBS subnetworks induced by cocaine. Violin plots comparing clustering coefficients, normalized clustering coefficients, and small-worldness between conditions for integrated and segregated states. The colored dots represent individual data, box limits indicate the 25th and 75th percentiles, horizontal lines mark the medians, white dots represent the mean, and whiskers cover the 1.5 interquartile range. The *p*-values were FDR-corrected using the Benjamini–Hochberg procedure; *p < 0.05 and **p < 0.001.

Next, we investigated the small-world properties of subnetworks (Figure 5). CUD exhibited higher clustering coefficients specifically in the integrated state (*F* = 15.31, *p* < 0.001), consistent with the pattern observed for local efficiency, as both metrics reflect specialized information processing capacity. However, when normalized by random networks, clustering coefficients were reduced in CUD during the segregated state (*F* = 8.95, *p* = 0.003). These results indicate that while CUD patients exhibited increased local connections, these connections may lack specificity and efficient organization. We did not find any significant differences in characteristic path length and normalized characteristic path length (Supplementary Figure 7). This discrepancy from global efficiency patterns may be attributed to differences in computational approaches, particularly given the exclusion of infinite distances. No significant differences were found in small-worldness for dynamic states (Figure 5). Interestingly, static functional network analysis revealed significantly reduced small-worldness in CUD (Supplementary Table 7), suggesting this alteration becomes more apparent when considering the entire scan duration. Validation using Brainnetome parcellation confirmed these findings (Supplementary Table 8), except that both the clustering coefficient and its normalized version showed significant differences across both states.

**Figure 6:**
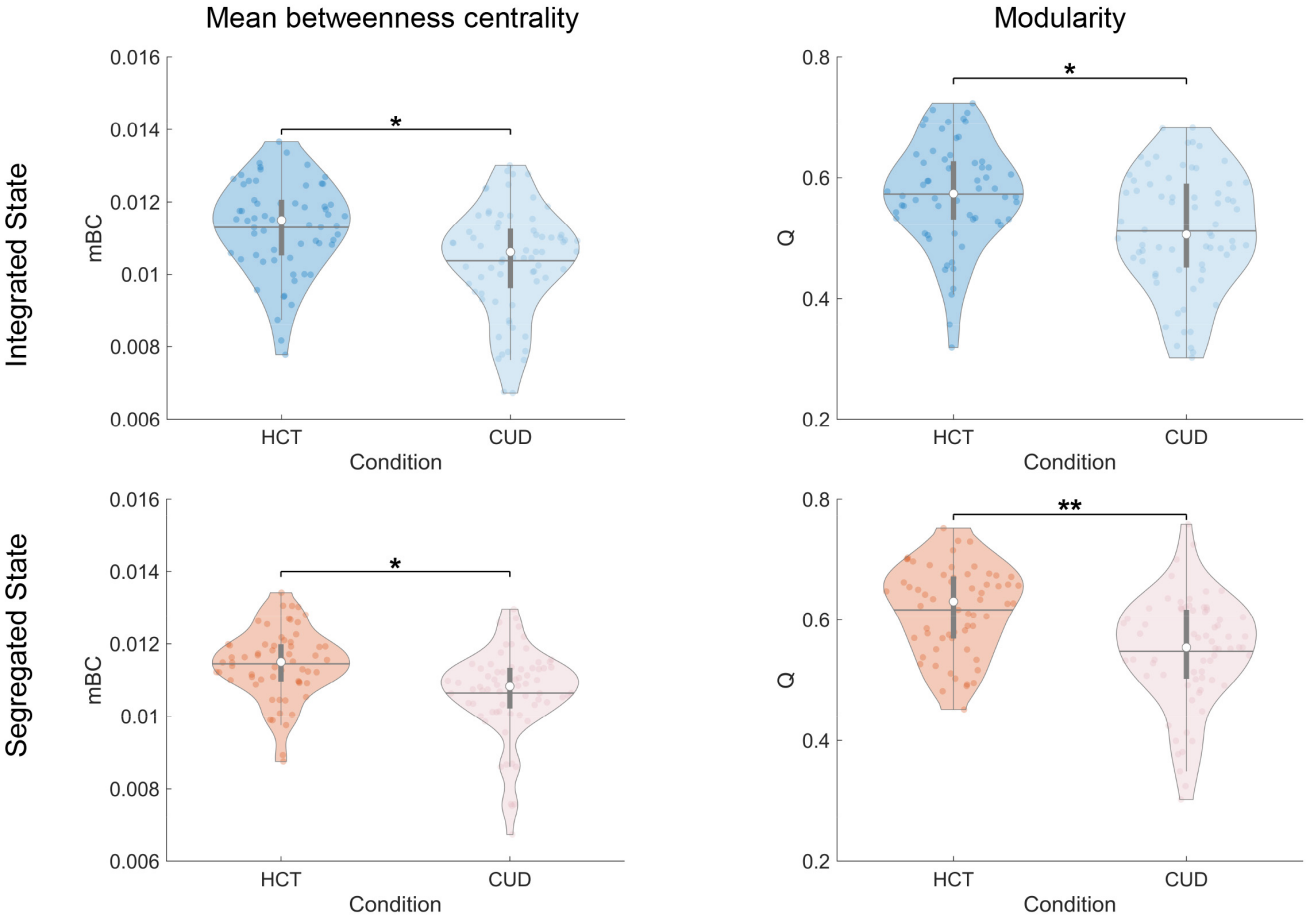
Cocaine-induced alterations in community structure. Violin plots showing mean betweenness centrality and modularity across conditions and states. The colored dots represent individual data, box limits indicate the 25th and 75th percentiles, horizontal lines mark the medians, white dots represent the mean, and whiskers cover the 1.5 interquartile range. The *p*-values were FDR-corrected using the Benjamini–Hochberg procedure;*p < 0.05 and **p < 0.001.

Finally, we examined community-related measures. Both mean betweenness centrality (integrated: *F* = 8.30, *p* = 0.005; segregated: *F* = 9.16, *p* = 0.003) and modularity (integrated: *F* = 7.79, *p* = 0.006; segregated: *F* = 13.34, *p* < 0.001) were significantly reduced in CUD compared to HCT (Figure 6). These cocaine-related alterations remained consistent across integrated and segregated states, suggesting uniform disruption in community structure across time. The Brainnetome atlas analysis replicated these findings (Supplementary Table 8).

### 3.4 Correlations with cocaine consumption patterns

In this section, we presented exploratory analyses of the associations between network properties in the CUD condition and cocaine consumption patterns to understand their physiological significance. To ensure reproducibility, we analyzed data using both the Schaefer-232 and Brainnetome atlases, focusing on correlations that remained significant across both parcellations (Supplementary Figure 8). The reported Pearson’s partial correlation coefficients and *p*-values were calculated using the Schaefer-232 atlas. We also performed the Shapiro–Wilk test for variables of identified significant associations and showed that the residuals of these measures did not deviate from the normal distribution (Supplementary Table 9), providing justification for this methodology.

**Figure 7:**
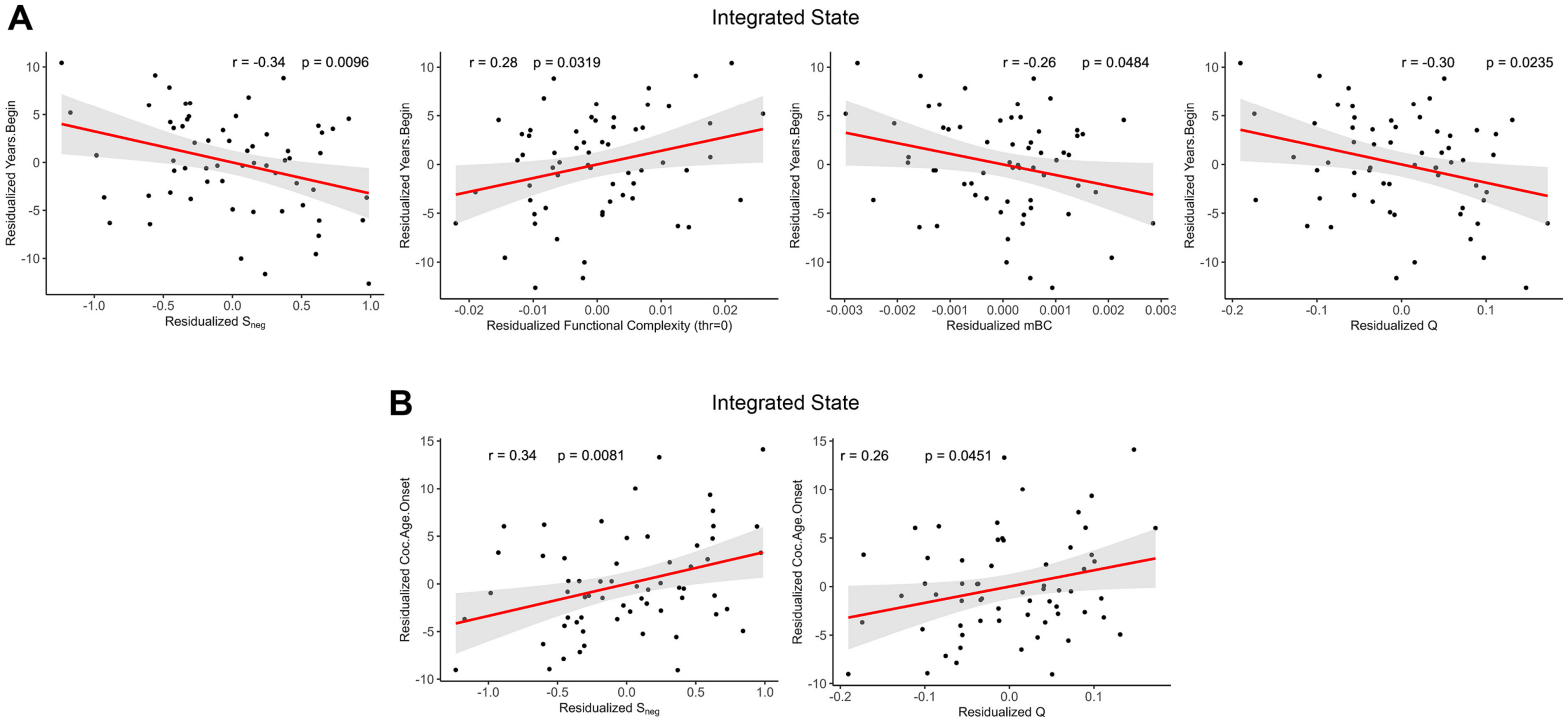
Relationships between cocaine consumption patterns and network properties in the integrated state after controlling age, sex, education, and mean framewise displacement. **(A)** Scatterplots of residualized years since the beginning of consumption of cocaine (Residualized Years.Begin) vs. residualized negative strength, functional complexity with threshold 0, betweenness centrality, and modularity. **(B)** Scatterplots of residualized cocaine age of onset (Residualized Coc.Age.Onset) vs. residualized negative strength and modularity. The r-values were Pearson’s partial correlation coefficients, and shaded areas represent 95% confidence.

We found reliable correlations exclusively in the integrated state (Figure 7). Specifically, years since the beginning of consumption of cocaine were negatively correlated with negative strength (r = −0.34, *p* = 0.0096), mean betweenness centrality (r = −0.26, *p* = 0.0484), and modularity (r = −0.30, *p* = 0.0235), while positively correlated with functional complexity at threshold 0 (r = 0.28, *p* = 0.0319). Cocaine age of onset showed positive correlations with negative strength (r = 0.34, *p* = 0.0081) and modularity (r = 0.26, *p* = 0.0451).

## 4 Discussion

In this study, we employed sliding-window analysis and cartographic profiling to characterize dynamic brain states with distinct topological configurations (integration vs. segregation) in healthy controls (HCT) and cocaine use disorder (CUD) groups. We found that CUD impacted both integrated and segregated states, with state-specific alterations in both connectivity and topological properties, providing novel insights into the dynamic neural mechanisms underlying cocaine addiction. Regarding functional connectivity, CUD significantly altered connectivity patterns involving the DMN, FPN, and SUB across both states. Besides, the integrated state showed distinct SMN connectivity alterations, and the segregated state exhibited pronounced FPN-SUB connectivity alterations. Notably, regional connectivity alterations showed significant correlations with spatial distributions of multiple neurotransmitter receptors, particularly in integrated states. Regarding network properties, CUD altered the positive–negative correlation balance, increased functional complexity (threshold 0), and reduced mean betweenness centrality and modularity across both states. While global efficiency, local efficiency, and clustering coefficient were increased in the integrated state, the normalized clustering coefficient was significantly reduced, particularly in segregated states, suggesting compromised functional organization. Additionally, CUD consistently exhibited reduced functional complexity with high thresholds in the segregated state. Notably, the network properties in the integrated state showed a significant correlation with cocaine consumption patterns, indicating their potential utility as quantifiable biomarkers for addiction development trajectories.

We found no significant differences between CUD and HCT groups in the integration–segregation dynamics, including fraction time, dwell time, total transitions, sample entropy, and Lempel–Ziv complexity of state sequences. These findings suggest that integration–segregation dynamics remain preserved in CUD, consistent with recent observations of maintained dynamics under LSD administration (Luppi et al., 2021a). Moreover, graph theory metrics at the whole-network level showed no significant differences between groups in either integrated or segregated states. This finding is consistent with previous studies that reported relatively preserved static functional network properties in both CUD and alcohol use disorder (AUD) compared to healthy controls (Sjoerds et al., 2017; Rasgado-Toledo et al., 2024; Morris et al., 2018). While the whole-network topology seemed resilient to CUD, our analyses revealed substantial alterations at the subnetwork level, indicating complex and nuanced effects of cocaine on brain network organization.

Using NBS, we identified significant connectivity alterations in CUD across both integrated and segregated states, demonstrating the pervasive impact of cocaine across time. The majority of affected connections exhibited increased connectivity strength in CUD, aligning with previous studies showing widespread functional hyperconnectivity in CUD patients in both static and dynamic networks (Cong et al., 2024; Wang et al., 2015). At the regional level, brain states showed pronounced connectivity alterations in the prefrontal cortex (PFC), precuneus, posterior cingulate cortex (PCC), and precentral ventral regions, highlighting the pivotal role of these regions in addictive behaviors. In line with our results, widespread PFC dysfunction was observed in CUD patients, associated with impaired task performance and elevated relapse risk (Goldstein and Volkow, 2011). Increased connectivity in PCC and precuneus was linked to heightened drug craving and impaired goal-directed behavior (Zhai et al., 2021). CUD patients also exhibited altered activity in the precentral gyri during multisensory tasks (Mayer et al., 2013). At the network level, both states showed significant functional reorganization between DMN-DAN, DMN-VAN, SUB-FPN, and within DMN. The DMN is a task-negative network crucial for self-reflection, emotional processing, and attentional allocation (Zhang and Volkow, 2019). The DMN-related alterations could reflect impaired capacity for introspective processes and negative emotion regulation, as well as enhanced attentional bias and craving for drug-related cues, facilitating cocaine-related memories (Zhang and Volkow, 2019; Cong et al., 2024; Zhai et al., 2021). Recent studies on cocaine addiction have reported altered connectivity between the cognitive control network and DMN, and within DMN, further supporting our findings (Cong et al., 2024; Xu et al., 2024). Besides, FPN governs higher-order cognitive control functions, including decision-making and task switching, while the subcortical network is essential for reward and motivation regulation. Abnormal connectivity between FPN and SUB could impair the ability to balance drug use and cognitive task demands. This result is supported by previous work examining craving or abstinence networks across various addictive disorders, including Internet gaming disorder, opioid use disorder, and cocaine use disorder (Zhou et al., 2022; Lichenstein et al., 2021; Yip et al., 2019). These investigations have consistently identified abnormal FPN-SUB connectivity, aligning with our result and suggesting a common neural mechanism underlying addiction disorders. We also identified state-specific functional reorganization. The integrated state showed characteristic changes between SMN-DMN and within SMN, suggesting that cocaine-induced disruptions in sensory processing and motor control predominantly emerge during integration. Previous fMRI research based on finger-tapping tasks has shown motor function abnormalities induced by chronic cocaine use (Lench et al., 2017). A recent study showed increased SMN-DMN connectivity across all brain states in CUD patients (Cong et al., 2024); our result expands this pattern by revealing its temporal preference in integration. The segregated state exhibited pronounced alterations between FPN-SUB, indicating that cognitive control deficits and aberrant reward processing in CUD predominantly manifest during network segregation (Feil et al., 2010). Our results, collectively, revealed state-general and state-specific neural changes across large-scale functional networks, providing insights into the potential role of integration and segregation in the pathophysiology of CUD.

We further explored the contributions of various neurotransmitter receptors and transporters to functional network reorganization. We identified consistent correlations for H3 and MOR receptors across both dynamic states. The mechanism of cocaine involves inhibiting dopamine, serotonin, and norepinephrine reuptake, triggering cascading effects across multiple receptor systems that collectively influence neuroplasticity and brain dynamics. Extensive evidence suggests that chronic cocaine exposure increases MOR receptor binding, which serves as a predictor for drug craving and treatment outcomes (Unterwald, 2001; Zubieta et al., 1996; Volkow, 2010). Moreover, H3 receptor antagonists demonstrated potential in addiction prevention by suppressing dopaminergic neuronal activity, thereby blocking dopamine-mediated feedforward mechanisms (Ellenbroek and Ghiabi, 2014). Notably, the integrated state revealed additional receptor associations, including mGluR5, CB1, M1, 5HT4, and 5HT6, suggesting the temporal preference in receptor-connectivity couplings. Extensive animal studies have shown strong associations between these receptors and cocaine addiction (Soria et al., 2005; Wang et al., 2013; Zacarias et al., 2012; Cunningham et al., 1992). In particular, the 5HT6 receptor, serving as a direct target of cocaine, modulates dopamine release, thereby enhancing sensitivity to the reinforcing properties of cocaine (Brodsky et al., 2016; Valentini et al., 2013). Recent research identified significant associations between 5HT4 and 5HT6 receptors and static connectivity alterations in CUD (Ricard et al., 2024), supporting our findings. This investigation also reported a significant positive correlation with the D2 receptor. Our results showed a similar positive correlation, although it did not achieve statistical significance, likely due to variations in data preprocessing and analytical approaches. Overall, these findings revealed potential links between receptor systems and dynamic functional networks, advancing our understanding of the neurophysiological characteristics of CUD and highlighting potential therapeutic targets.

Given the extensive functional connectivity alterations identified by NBS, we conducted a graph theory analysis on the NBS subnetworks. We found a common pattern in which CUD exhibited increased positive strength and decreased negative strength compared to HCT. In typical resting-state brains, DMN shows widespread negative correlations with task-positive regions; the shifted balance between positive and negative correlation may suggest impaired inhibitory control and cognitive dysfunction (Zhai et al., 2021). We also found that CUD patients exhibited higher global efficiency, local efficiency, and clustering coefficients, particularly in the integrated state, and greater functional complexity at threshold 0 in both states. These patterns suggest that the brain tends to establish richer connections to support efficient global and local information communication. These results may reflect neural pathological alterations associated with chronic cocaine use, where additional resources are allocated either to mitigate cognitive decline or to reinforce addiction-related circuits (Dayan, 2009). Notably, we observed a reliable decrease in functional complexity at higher thresholds in the segregated states, which contrasts with the result at threshold 0, suggesting that the diversity of efficient information transfer remains impaired despite compensation. Additionally, we did not observe similar phenomena in the integrated or static state, highlighting the importance of dynamic analysis. Interestingly, CUD showed significantly lower normalized clustering coefficients than HCT, indicating a lack of specific and effective network organization. We did not find differences in small-worldness between the two states. However, static network analysis revealed a reduction in small-worldness in CUD. This result is consistent with findings in heroin (Jiang et al., 2013), cocaine (Wang et al., 2015), and methamphetamine addiction studies (Luo et al., 2024), reflecting dysfunction in local specialization and global integration. Our analysis suggests that the small-worldness alterations in CUD tend to reflect in time-averaged characteristics rather than in time-resolved patterns. Finally, we found that CUD showed a decreased mean betweenness centrality and modularity in both integrated and segregated states. Nodes with high betweenness centrality usually serve as connector hubs between functionally specialized modules, facilitating global information integration (Sporns et al., 2007; GeethaRamani and Sivaselvi, 2014). The reduced hub functionality coupled with modular structure disruption indicates compromised capabilities of brain networks in both specialized processing and global information integration. Overall, our results provided network topological changes from the dynamic integration and segregation perspective, revealing widespread network communication abnormalities induced by CUD.

Our analysis revealed significant associations between network metrics and cocaine consumption patterns, providing critical insights into the potential neural alterations associated with chronic cocaine exposure. Specifically, years since the beginning of cocaine use negatively correlated with negative strength, betweenness centrality, and modularity while positively correlated with functional complexity at threshold 0. Conversely, cocaine age of onset exhibited positive relationships with negative strength and modularity. Notably, consistent associations were observed only in the integrated state, indicating its superior sensitivity in capturing systematic alterations in brain functional architecture linked to long-term cocaine use. Furthermore, the robust associations of negative strength and modularity with cocaine consumption patterns suggest their potential utility as neuroimaging biomarkers for tracking disease progression and treatment outcomes, potentially facilitating early intervention strategies and personalized therapeutic approaches.

These findings could be synthesized into a unified hypothetical framework that highlights that the pathological brain network disruption and reorganization induced by chronic cocaine exposure is primarily characterized by functional hyperconnectivity (CUD > HCT) (Ricard et al., 2024; Cong et al., 2024; Wang et al., 2015). We validated and extended this hypothetical model from a dynamic integration and segregation perspective. Our results demonstrated that CUD-induced dynamic connectivity alterations manifest predominantly as enhanced functional connectivity patterns. On the one hand, these hyperconnected pathways exhibited stronger associations with regional receptor density distributions compared to hypoconnected connections (Supplementary Tables 10–13), emphasizing the fundamental neurophysiological constraints underlying network reorganization. The integrated state showed more receptor-function couplings, indicating the potential therapeutic interventions through selective receptor agonists or inhibitors to mitigate addiction-related neural signatures. On the other hand, the pervasive enhancement of functional connectivity could drive systematic alterations in global network architecture, leading to increased positive strength, efficiency-related metrics, and functional complexity at threshold 0, concurrent with decreased negative strength. The non-specific nature and disorder of such functional reorganization also disrupted the original brain network structure, resulting in reduced functional complexity at threshold 0.3, and decreased mean betweenness centrality and modularity. The topological alterations in the integrated state were consistently linked to cocaine consumption patterns, suggesting their utility as potential neuroimaging biomarkers for tracking disease trajectory and treatment response. While this paragraph focuses on hyperconnectivity, we also observed significant hypoconnectivity. Future research should further explore the interplay and distinctions between hyperconnectivity and hypoconnectivity to gain a more comprehensive understanding of the neural impact of CUD. Our study has several limitations. First, our analysis was confined to resting-state fMRI data of CUD patients meeting specific inclusion criteria of the dataset; future research should examine network dynamics across different stages of cocaine use, including acute intake and withdrawal periods. Meanwhile, incorporating task-based paradigms would provide complementary insights into the CUD’s neural mechanisms, particularly regarding reward processing and “cognitive control”. In addition, global signal regression (GSR) is a common preprocessing step in fMRI data analysis that helps remove physiological noise and motion artifacts (Ricard et al., 2024; Chan et al., 2021). However, recent studies suggest that the global signal contains meaningful neural activity components, such as arousal fluctuations and vascular signals (Liu et al., 2017; Huber et al., 2024). GSR may lead to the loss of meaningful signals. Future research should develop more refined analytical frameworks to account for connectivity differences while incorporating the global signal. Finally, we focused on global network properties aligned with previous research (Luppi et al., 2021a, 2021b); future investigations should extend to node-level analyses to identify regional contributions to network dysfunction.

## Data Availability

Publicly available datasets were analyzed in this study. This data can be found at: https://openneuro.org/datasets/ds003346/versions/1.1.2.
