# Supplementary material for: Altered integrated and segregated states in cocaine use disorder
Source: Front Neurosci. 2025 Apr 9;19:1572463. doi: 10.3389/fnins.2025.1572463 (PMC12014740; doi:10.3389/fnins.2025.1572463)
Supplement: Supplementary file 1 [file Data_Sheet_1.pdf]

Supplementary Material

1 Supplementary Information

1.1 Graph theoretical measures

1.1.1 Strength and efficiency metrics

Network measures related to edge strength and efficiency included positive strength, negative strength, global efficiency, local efficiency, and functional complexity. Positive strength was calculated as

$$S_{pos} = \frac{1}{n}\sum_{i}\sum_{j\in N, j\neq i} w_{ij}^{+}$$

where w_{ij}^{+} represents the positive weight between the regions i and j, n is the number of regions, and N is the set of all regions. It quantifies the mean strength of coordinated information transfer across nodes. Negative strength was calculated as

$$S_{neg} = \frac{1}{n}\sum_{i}\sum_{j\in N, j\neq i} w_{ij}^{-}$$

where w_{ij}^{-} represents the negative weight between the regions i and j. It represents the overall negative correlation in the brain. Global efficiency was calculated as

$$E_{global} = \frac{1}{n}\sum_{i}\frac{1}{(n-1)}\sum_{j\in N, j\neq i}\frac{1}{d_{ij}}$$

where d_{ij} represents the shortest path length between the regions i and j. Local efficiency was calculated as

$$E_{local} = \frac{1}{n}\sum_{i} E_{global}(G_i)$$

where G_i represents the subgraph of the node i and its neighboring nodes. High local efficiency enables specialized processing within neural subsystems, while high global efficiency supports efficient information flow across the whole-brain network. The functional complexity was calculated as

$$\text{Functional Complexity} = 1 - \frac{1}{C_m}\sum_{\mu=1}^{m}\left|p_{\mu}(r) - \frac{1}{m}\right|$$

where $C_m = 2\frac{m-1}{m}$ represents the normalization coefficient, and $p_\mu(r)$ is the probability distribution of connection weight in m bins. It quantifies the similarity between the empirical connectivity distribution and a uniform distribution, thereby reflecting the network's functional diversity[1]. We excluded negative connections and considered only positive couplings when calculating this measure. Additionally, we modified the threshold to 0.3 to analyze functional complexity under strong couplings. To validate the robustness of our findings, we conducted additional analyses using thresholds of 0.26, 0.28, 0.32, and 0.34.

1.1.2 Small-world properties

Small-world properties are related to the clustering coefficient, characteristic path length, normalized clustering coefficient, normalized characteristic path length, and small-worldness. Since these metrics relied on path calculations, only positive weights were considered. The clustering coefficient was calculated as

$$C = \frac{1}{n}\sum_i \frac{1}{k_i(k_i-1)} \sum_{j,k\in N} (w_{ij}^+ w_{jk}^+ w_{ki}^+)^{\frac{1}{3}}$$

where $w_{ij}^+, w_{jk}^+, w_{ki}^+$ are positive weights between the regions i, j and k, and k_i is the positive nodal strength of region i. It represents the mean tightness of connections between nodes and their neighbors. The characteristic path length was computed as

$$L = \frac{1}{n}\sum_i \frac{1}{n-1} \sum_{j\in N, j\neq i} d_{ij}$$

where d_{ij} represents the shortest path length between the regions i and j. Infinite distances were excluded in the calculation of L. For each empirical network, we generated 100 rewired null models that preserved degree distributions. These null models underwent the same graph analysis, generating the mean values as C_{rand} and L_{rand}. The normalized clustering coefficient and characteristic path length were defined as $nC = \frac{C}{C_{rand}}$ and $nL = \frac{L}{L_{rand}}$, representing their performance relative to random networks. The small-worldness was calculated as

$$SW = \frac{nC}{nL}$$

High small-worldness indicates the large clustering coefficient and relatively short path length, which is observed in many real-world networks including the brain, reflecting a balance between local and global information processing[2].

1.1.3 Community structure

We used the mean betweenness centrality and modularity to characterize the community structure of the network. The mean betweenness centrality was calculated as

$$mBC = \frac{1}{n}\sum_{i}\frac{1}{(n-1)(n-2)}\sum_{j,k\in N, j\neq k\neq i}\frac{\sigma_{jk}(i)}{\sigma_{jk}}$$

where σ_{jk} is the number of shortest paths between the regions j and k, and $\sigma_{jk}(i)$ is the number of shortest paths between the regions j and k that passed through region i. Betweenness centrality indirectly reflects community organization, making it a critical component in network-based community detection methodologies[3]. We used the average of node-level metrics to examine system-wide topological reorganization. Modularity Q was defined in the main text. It directly evaluated the community partition quality and characterized the segregation of network information processing.

2 Supplementary Figures and Tables

2.1 Supplementary Figures

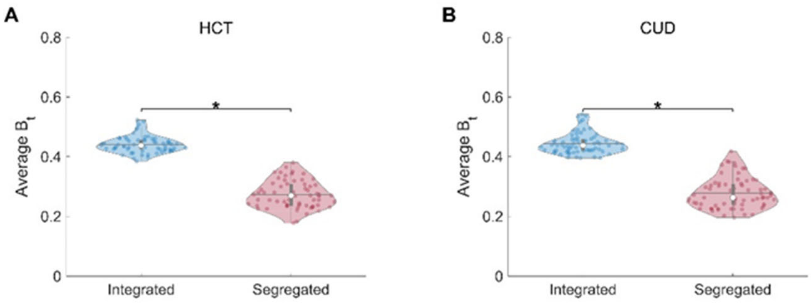

Supplementary Figure 1. Significant differences in the mean participation coefficient between integrated and segregated states for CUD and HCT conditions. Colored dots represent individual data, box limits indicate the 25th and 75th percentiles, horizontal lines mark the medians, white dots represent the mean, and whiskers cover the 1.5 interquartile range. The p values were FDR-corrected using Benjamini-Hochberg procedure; * $p < 0.001$.

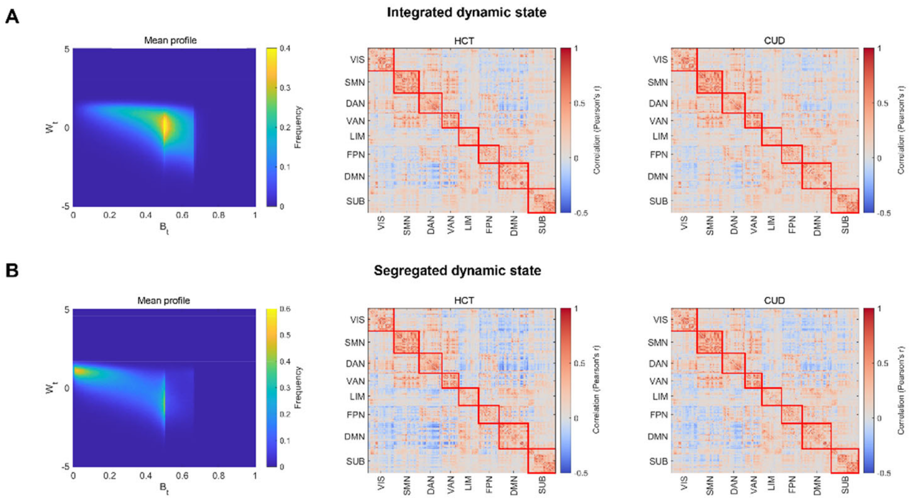

Supplementary Figure 2. The mean profiles and centroids of the HCT and CUD groups derived using the Brainnetome atlas. (A) The integrated state. (B) The segregated state.

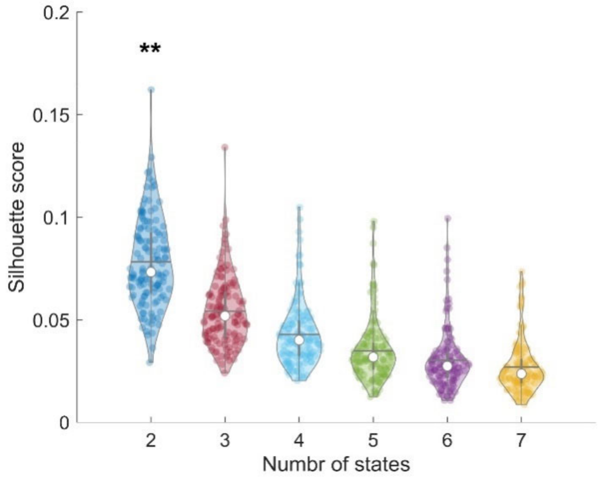

Supplementary Figure 3. Silhouette scores for k-means clustering with cluster number ranging from 2 to 7. ** represents that K = 2 has the highest scores compared to other cluster number. Statistical significance was examined using paired t-tests.

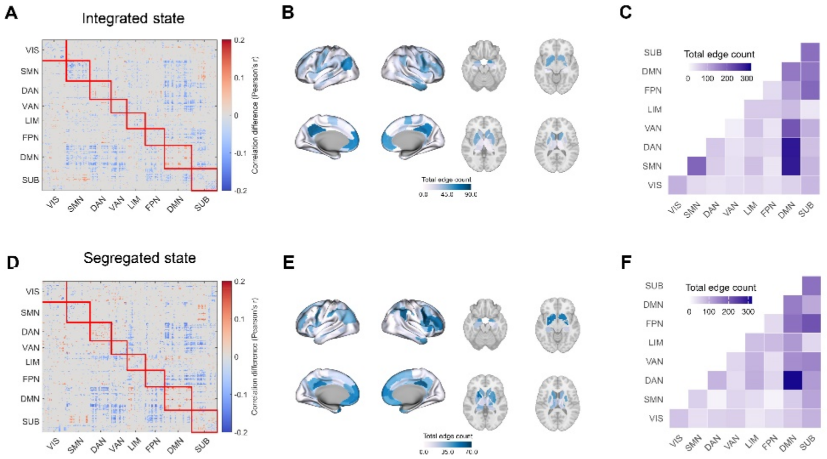

Supplementary Figure 4. Cocaine-induced changes in dynamic functional connectivity based on the Brainnetome atlas. (A) (D) Network-based statistic (NBS) results for integrated and segregated states (red: HCT > CUD; blue: CUD > HCT). Brain regions are organized according to the Yeo 7 networks and subcortical structures (VIS: visual network, SMN: sensorimotor network, DAN: dorsal attention network, VAN: ventral attention network, FPN: frontoparietal network, DMN: default mode network, SUB: subcortical structures). (B) (E) Visualization of the regional counts of significant edges. (C) (F) Network-level significant edge counts for integrated and segregated states.

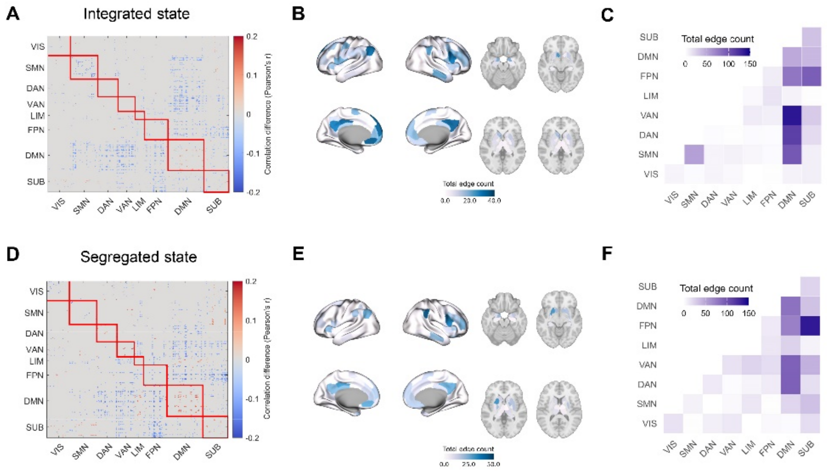

Supplementary Figure 5. Changes in dynamic functional connectivity based on a strict component forming threshold (0.01). (A) (D) Network-based statistic (NBS) results for integrated and segregated states (red: HCT > CUD; blue: CUD > HCT). Brain regions are organized according to the Yeo 7 networks and subcortical structures (VIS: visual network, SMN: sensorimotor network, DAN: dorsal attention network, VAN: ventral attention network, FPN: frontoparietal network, DMN: default mode network, SUB: subcortical structures). (B) (E) Visualization of the regional counts of significant edges. (C) (F) Network-level significant edge counts for integrated and segregated states.

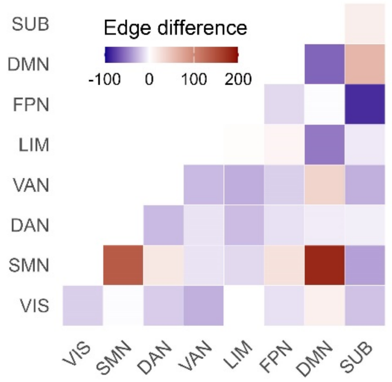

Supplementary Figure 6. Differences in the network-level significant edge count between integrated and segregated states (red: integrated > segregated; blue: segregated > integrated).

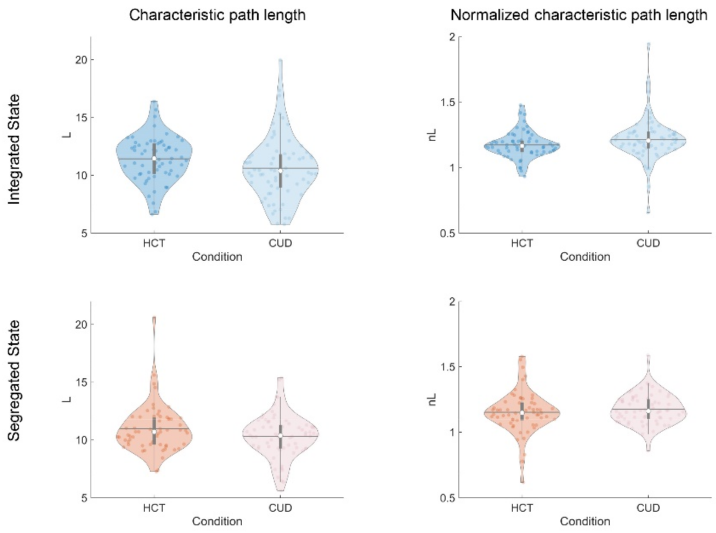

Supplementary Figure 7. Characteristic path length and normalized characteristic path length in integrated and segregated states. Colored dots represent individual data, box limits indicate the 25th and 75th percentiles, horizontal lines mark the medians, white dots represent the mean, and whiskers cover the 1.5 interquartile range. No differences are found between HCT and CUD groups.

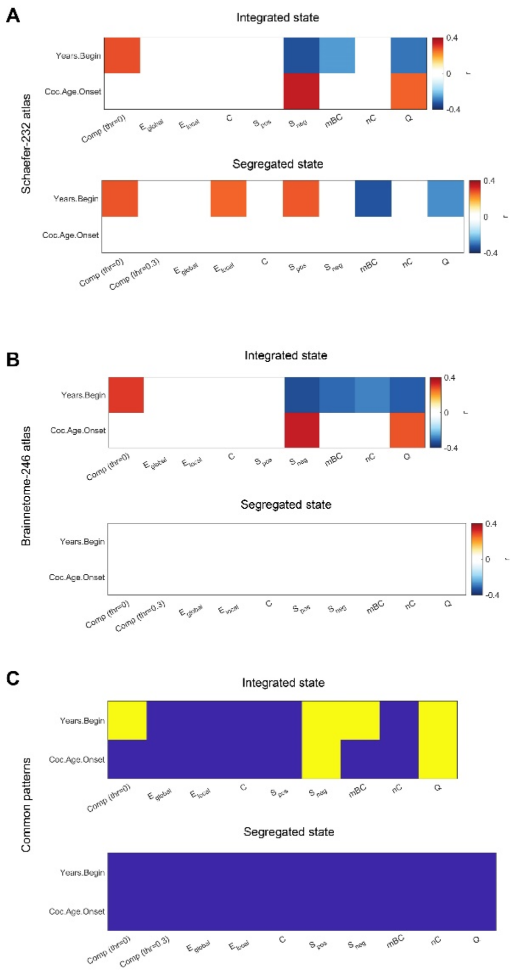

Supplementary Figure 8. Significant Pearson's partial correlations between network properties and cocaine consumption patterns in the CUD group for both integrated and segregated states. (A) Measures derived from the Schaefer-232 atlas. (B) Measures derived from the Brainnetome 246-region atlas. (C) Yellow cells indicate significant correlations across the two parcellations. Years.Begin: years since beginning of consumption of cocaine; Coc.Age.Onset: cocaine age of onset; Comp (thr = 0): functional complexity at threshold 0; Comp (thr = 0.3): functional complexity at threshold 0.3; E_{global}: global efficiency; E_{local}: local efficiency; C: clustering coefficient; nC: normalized clustering coefficient; S_{pos}: positive strength; S_{neg}: negative strength; mBC: mean betweenness centrality; Q: modularity.

2.2 Supplementary Tables

Supplementary Table 1. Differences in subnetwork properties between CUD and healthy controls identified through ANCOVA across integrated and segregated states (Schaefer-232 parcellation).

Measure	Mean difference	F-stat	p-value
State sequence			
Sample Entropy	-0.0164	1.2004	0.2755
Lempel-Ziv Complexity	-0.0818	1.2160	0.2724
Total Transitions	-2.1575	1.2635	0.2633
Integrated dynamic state			
Fraction Time	0.0047	0.4285	0.5140
Dwell Time	0.4469	0.0054	0.9417
Global efficiency (E_{global})	-0.0135	15.7139	0.0001
Local efficiency (E_{local})	-0.0127	5.5439	0.0202
Positive Strength (S_{pos})	-0.7137	9.8356	0.0022
Negative Strength (S_{neg})	0.5837	13.9675	0.0003
Functional Complexity (thr = 0)	-0.0099	14.9353	0.0002
Functional Complexity (thr = 0.26)	0.0033	0.3995	0.5286
Functional Complexity (thr = 0.28)	0.0011	0.1248	0.7245
Functional Complexity (thr = 0.3)	-0.0013	0.0027	0.9585
Functional Complexity (thr = 0.32)	-0.0040	0.0973	0.7556
Functional Complexity (thr = 0.34)	-0.0065	0.4493	0.5040
Clustering Coeff (C)	-0.0053	15.3058	0.0002
Normalized Clustering Coeff (nC)	0.1548	2.7527	0.0997
Characteristic Path Length (L)	0.8001	0.7132	0.4001
Normalized Characteristic Path Length (nL)	-0.0829	1.2782	0.2605

small-worldness (SW)	0.4750	2.7825	0.0980
Mean Betweenness Centrality (mBC)	0.0009	8.3033	0.0047
Modularity (Q)	0.0614	7.7895	0.0061
Segregated dynamic state			
Fraction Time	-0.0047	0.4285	0.5140
Dwell Time	0.2022	1.0577	0.3058
Global efficiency (E_{global})	-0.0075	2.0231	0.1576
Local efficiency (E_{local})	-0.0035	3.0771	0.0820
Positive Strength (S_{pos})	-0.4739	7.7557	0.0062
Negative Strength (S_{neg})	0.6609	13.0897	0.0004
Functional Complexity (thr = 0)	-0.0084	16.5274	0.0001
Functional Complexity (thr = 0.26)	0.0324	14.5550	0.0002
Functional Complexity (thr = 0.28)	0.0306	14.3116	0.0002
Functional Complexity (thr = 0.3)	0.0291	14.1536	0.0003
Functional Complexity (thr = 0.32)	0.0270	13.0083	0.0006
Functional Complexity (thr = 0.34)	0.0238	10.8991	0.0013
Clustering Coeff (C)	0.0006	0.1070	0.7442
Normalized Clustering Coeff (nC)	0.2492	8.9541	0.0034
Characteristic Path Length (L)	7.1461	1.7485	0.1886
Normalized Characteristic Path Length (nL)	0.4576	1.4630	0.2289
small-worldness (SW)	0.1703	1.3358	0.2501
Mean Betweenness Centrality (mBC)	0.0008	9.1597	0.0030
Modularity (Q)	0.0688	13.3400	0.0004

Supplementary Table 2. Cross-atlas validation: Spearman's correlations between receptor density distributions (same as in the main text) and regional connectivity alterations in the integrated state using the Brainnetome atlas. P-values, derived from the spin test for cortical regions and random shuffling for subcortical regions, are FDR-corrected.

Source	Space	Neurotransmitter	Receptor	Corr	p-value
savli2012	MNI152	serotonin	5HT1a	0.032	0.9071
gallezot2010	MNI152	serotonin	5HT1b	0.300	0.0053
beliveau2017	fsaverage	serotonin	5HT2a	0.095	0.4057
beliveau2017	fsaverage	serotonin	5HT4	0.225	0.0441
radnakrishnan2018	MNI152	serotonin	5HT6	0.276	0.0052
beliveau2017	fsaverage	serotonin	5HTT	-0.014	0.9071
kantonen2020	MNI152	opioid	MOR	0.274	0.0285
ding2010	MNI152	norepinephrine	NET	0.022	0.9071
gallezot2017	MNI152	histamine	H3	0.445	0.0010
smart2019	MNI152	glutamate	mGluR5	0.281	0.0051
dukart2018	MNI152	gaba	GABAa	0.021	0.9071
norgaard2021	fsaverage	gaba	GABAa-bz	0.009	0.9071
kaller2017	MNI152	dopamine	D1	0.228	0.0515
smith2017	MNI152	dopamine	D2	0.036	0.9071
sasaki2012	MNI152	dopamine	DAT	0.027	0.9071
normandin2015	MNI152	cannabinoid	CB1	0.346	0.0010
hillmer2016	MNI152	acetylcholine	A4B2	0.210	0.1213
naganawa2020	MNI152	acetylcholine	M1	0.245	0.0071
aghourian2017	MNI152	acetylcholine	VAChT	0.253	0.0071

Supplementary Table 3. Reproducibility tests: Spearman's correlations between receptor density distributions (additional maps) and regional connectivity alterations in the integrated state using the Schaefer-232 atlas. P-values are derived from the spin test for cortical regions and random shuffling for subcortical regions.

Source	Space	Neurotransmitter	Receptor	Corr	p-value
sandiego2015	MNI152	dopamine	D2	0.084	0.5599
jaworska2020	MNI152	dopamine	D2	0.086	0.5585
bedard2019	MNI152	acetylcholine	VAChT	0.120	0.2483
tuominen	MNI152	acetylcholine	VAChT	0.091	0.2987
laurikainen2018	MNI152	cannabinoid	CB1	0.332	0.0003
dubois2015	MNI152	glutamate	mGluR5	0.291	0.0024
rosaneto	MNI152	glutamate	mGluR5	0.228	0.0164
hesse2017	MNI152	norepinephrine	NET	0.029	0.7204
turtonen2020	MNI152	opioid	MOR	0.347	0.0004
beliveau2017	fsaverage	serotonin	5HT1a	0.088	0.5273
beliveau2017	fsaverage	serotonin	5HT-1b	0.005	0.9531
savli2012	MNI152	serotonin	5HT-1b	0.187	0.0480
savli2012	MNI152	serotonin	5HT2a	0.061	0.5751
savli2012	MNI152	serotonin	5HTT	-0.019	0.8833
fazio2016	MNI152	serotonin	5HTT	0.067	0.5265

Supplementary Table 4. Cross-atlas validation: Spearman's correlations between receptor density distributions (same as in the main text) and regional connectivity alterations in the segregated state using the Brainnetome atlas. P-values, derived from the spin test for cortical regions and random shuffling for subcortical regions, are FDR-corrected.

Source	Space	Neurotransmitter	Receptor	Corr	p-value
savli2012	MNI152	serotonin	5HT1a	-0.012	0.9415
gallezot2010	MNI152	serotonin	5HT1b	0.192	0.1718

beliveau2017	fsaverage	serotonin	5HT2a	0.011	0.9415
beliveau2017	fsaverage	serotonin	5HT4	0.167	0.2551
radnakrishnan2018	MNI152	serotonin	5HT6	0.155	0.1718
beliveau2017	fsaverage	serotonin	5HTT	0.041	0.8442
kantonen2020	MNI152	opioid	MOR	0.434	0.0019
ding2010	MNI152	norepinephrine	NET	-0.115	0.5213
gallezot2017	MNI152	histamine	H3	0.378	0.0038
smart2019	MNI152	glutamate	mGluR5	0.157	0.2584
dukart2018	MNI152	gaba	GABAa	-0.075	0.6702
norgaard2021	fsaverage	gaba	GABAa-bz	-0.122	0.5045
kaller2017	MNI152	dopamine	D1	0.239	0.0813
smith2017	MNI152	dopamine	D2	0.147	0.5045
sasaki2012	MNI152	dopamine	DAT	0.069	0.5411
normandin2015	MNI152	cannabinoid	CB1	0.264	0.0813
hillmer2016	MNI152	acetylcholine	A4B2	0.287	0.0813
naganawa2020	MNI152	acetylcholine	M1	0.115	0.3258
aghourian2017	MNI152	acetylcholine	VAChT	0.170	0.3258

Supplementary Table 5. Reproducibility tests: Spearman's correlations between receptor density distributions (additional maps) and regional connectivity alterations in the segregated state using the Schaefer-232 atlas. P-values are derived from the spin test for cortical regions and random shuffling for subcortical regions.

Source	Space	Neurotransmitter	Receptor	Corr	p-value
sandiego2015	MNI152	dopamine	D2	0.195	0.2054
jaworska2020	MNI152	dopamine	D2	0.178	0.2421
bedard2019	MNI152	acetylcholine	VAChT	0.175	0.1984

tuominen	MNI152	acetylcholine	VAChT	0.104	0.4594
laurikainen2018	MNI152	cannabinoid	CB1	0.198	0.0715
dubois2015	MNI152	glutamate	mGluR5	0.192	0.0539
rosaneto	MNI152	glutamate	mGluR5	0.168	0.0847
hesse2017	MNI152	norepinephrine	NET	-0.089	0.2750
turtonen2020	MNI152	opioid	MOR	0.424	0.0001
beliveau2017	fsaverage	serotonin	5HT1a	0.112	0.4025
beliveau2017	fsaverage	serotonin	5HT-1b	0.023	0.8507
savli2012	MNI152	serotonin	5HT-1b	0.068	0.5351
savli2012	MNI152	serotonin	5HT2a	-0.025	0.8544
savli2012	MNI152	serotonin	5HTT	0.110	0.4523
fazio2016	MNI152	serotonin	5HTT	0.182	0.1718

Supplementary Table 6. Differences in whole-network properties between CUD and healthy controls identified through ANCOVA across integrated and segregated states (Schaefer-232 parcellation).

Measure	Mean difference	F-stat	p-value
State sequence			
Sample Entropy	-0.0164	1.2004	0.2755
Lempel-Ziv Complexity	-0.0818	1.2160	0.2724
Total Transitions	-2.1575	1.2635	0.2633
Integrated dynamic state			
Fraction Time	0.0047	0.4285	0.5140
Dwell Time	0.4469	0.0054	0.9417
Global efficiency (E_{global})	-0.0055	0.0294	0.8642
Local efficiency (E_{local})	-0.0055	0.0371	0.8476

Positive Strength (S_{pos})	-2.1415	0.1704	0.6805
Negative Strength (S_{neg})	0.8176	0.1599	0.6899
Functional Complexity (thr = 0)	-0.0026	0.0895	0.7653
Functional Complexity (thr = 0.26)	-0.0002	0.1342	0.7148
Functional Complexity (thr = 0.28)	-0.0003	0.1885	0.6649
Functional Complexity (thr = 0.3)	-0.0005	0.2723	0.6028
Functional Complexity (thr = 0.32)	-0.0012	0.4491	0.5041
Functional Complexity (thr = 0.34)	-0.0016	0.5232	0.4709
Clustering Coeff (C)	-0.0069	0.0681	0.7946
Normalized Clustering Coeff (nC)	0.0117	0.0000	0.9994
Characteristic Path Length (L)	0.0707	0.0014	0.9699
Normalized Characteristic Path Length (nL)	0.0086	0.0061	0.9376
small-worldness (SW)	0.0035	0.0000	0.9949
Mean Betweenness Centrality (mBC)	0.0001	0.0717	0.7894
Modularity (Q)	0.0116	0.0243	0.8764
Segregated dynamic state			
Fraction Time	-0.0047	0.4285	0.5140
Dwell Time	0.2022	1.0577	0.3058
Global efficiency (E_{global})	-0.0029	0.0088	0.9254
Local efficiency (E_{local})	-0.0005	0.0496	0.8242
Positive Strength (S_{pos})	-1.4019	0.1902	0.6636
Negative Strength (S_{neg})	1.2021	0.14544	0.7036
Functional Complexity (thr = 0)	0.0069	0.6230	0.4315
Functional Complexity (thr = 0.26)	0.0061	0.5396	0.4641

Functional Complexity (thr = 0.28)	0.0052	0.3995	0.5286
Functional Complexity (thr = 0.3)	0.0044	0.2851	0.5944
Functional Complexity (thr = 0.32)	0.0038	0.2151	0.6437
Functional Complexity (thr = 0.34)	0.0034	0.1745	0.6769
Clustering Coeff (C)	-0.0015	0.0000	0.9952
Normalized Clustering Coeff (nC)	0.0249	0.0783	0.7801
Characteristic Path Length (L)	0.0512	0.0585	0.8094
Normalized Characteristic Path Length (nL)	0.0239	1.3910	0.2406
small-worldness (SW)	0.0023	0.2121	0.6460
Mean Betweenness Centrality (mBC)	0.0001	0.1802	0.6720
Modularity (Q)	0.0204	0.4145	0.5210

Supplementary Table 7. Differences in static functional subnetwork properties between CUD and healthy controls identified through ANCOVA (Schaefer-232 parcellation).

Measure	Mean difference	F-stat	p-value
Static functional connectivity			
Global efficiency (E_{global})	-0.0146	17.7553	0.0000
Local efficiency (E_{local})	-0.0144	7.6460	0.0066
Positive Strength (S_{pos})	-0.8996	12.9898	0.0005
Negative Strength (S_{neg})	0.7279	13.5413	0.0004
Functional Complexity (thr = 0)	-0.0118	17.2690	0.0000
Functional Complexity (thr = 0.26)	0.0095	1.2146	0.2727
Functional Complexity (thr = 0.28)	0.0080	0.8761	0.3512
Functional Complexity (thr = 0.3)	0.0057	0.4335	0.5116
Functional Complexity (thr = 0.32)	0.0034	0.1200	0.7296
Functional Complexity (thr = 0.34)	0.0008	0.0000	0.9974

Clustering Coeff (C)	-0.0049	11.8925	0.0008
Normalized Clustering Coeff (nC)	0.2694	5.0556	0.0264
Characteristic Path Length (L)	0.4605	0.0007	0.9784
Normalized Characteristic Path Length (nL)	-0.1107	3.2984	0.0719
small-worldness (SW)	0.3611	7.5413	0.0070
Mean Betweenness Centrality (mBC)	0.0009	8.07278	0.0053
Modularity (Q)	0.0772	12.9368	0.0005

Supplementary Table 8. Cross-atlas validation: differences in subnetwork properties between CUD and healthy controls identified through ANCOVA across integrated and segregated states (Brainnetome-246 parcellation).

Measure	Mean difference	F-stat	p-value
State sequence			
Sample Entropy	-0.0083	0.3848	0.5363
Lempel-Ziv Complexity	-0.0521	0.3483	0.5562
Total Transitions	-1.4269	0.5184	0.4729
Integrated dynamic state			
Fraction Time	0.0032	0.0287	0.8658
Dwell Time	1.0961	0.8494	0.3586
Global efficiency (E_{global})	-0.0119	14.5344	0.0002
Local efficiency (E_{local})	-0.0126	4.0149	0.0474
Positive Strength (S_{pos})	-0.6841	8.3992	0.0045
Negative Strength (S_{neg})	0.5671	13.3043	0.0004
Functional Complexity (thr = 0)	-0.0090	13.1883	0.0004
Functional Complexity (thr = 0.26)	0.0014	0.0023	0.9620
Functional Complexity (thr = 0.28)	-0.0014	0.1073	0.7439

Functional Complexity (thr = 0.3)	-0.0035	0.3056	0.5814
Functional Complexity (thr = 0.32)	-0.0058	0.6932	0.4067
Functional Complexity (thr = 0.34)	-0.0081	1.1737	0.2808
Clustering Coeff (C)	-0.0053	13.9756	0.0003
Normalized Clustering Coeff (nC)	0.2392	3.9930	0.0480
Characteristic Path Length (L)	-0.9141	0.1530	0.6964
Normalized Characteristic Path Length (nL)	-0.2561	0.7300	0.3946
small-worldness (SW)	0.3922	4.0092	0.0475
Mean Betweenness Centrality (mBC)	0.0010	8.0514	0.0054
Modularity (Q)	0.0638	8.2875	0.0047
Segregated dynamic state			
Fraction Time	-0.0032	0.0287	0.8658
Dwell Time	0.2717	1.2744	0.2612
Global efficiency (E_{global})	-0.0119	11.4910	0.0010
Local efficiency (E_{local})	-0.0095	9.6804	0.0023
Positive Strength (S_{pos})	-0.6516	13.3088	0.0004
Negative Strength (S_{neg})	0.6442	14.8786	0.0002
Functional Complexity (thr = 0)	-0.0083	19.1423	0.0000
Functional Complexity (thr = 0.26)	0.0196	6.7133	0.0108
Functional Complexity (thr = 0.28)	0.0197	7.4461	0.0073
Functional Complexity (thr = 0.3)	0.0196	7.8192	0.0060
Functional Complexity (thr = 0.32)	0.0187	7.5835	0.0068
Functional Complexity (thr = 0.34)	0.0171	6.7648	0.0105
Clustering Coeff (C)	-0.0030	5.6697	0.0189
Normalized Clustering Coeff (nC)	0.2446	7.8426	0.0060

Characteristic Path Length (L)	-3.8537	0.9811	0.3240
Normalized Characteristic Path Length (nL)	-0.5018	1.2295	0.2698
small-worldness (SW)	0.1633	1.1842	0.2787
Mean Betweenness Centrality (mBC)	0.0010	13.5800	0.0003
Modularity (Q)	0.0659	13.9775	0.0003

Supplementary Table 9. Results of the Shapiro-Wilk test for the residuals of cocaine consumption patterns and network metrics. Years.Begin: years since beginning of consumption of cocaine; Coc.Age.Onset: cocaine age of onset; S_{neg}: negative strength; Comp (thr = 0): functional complexity at threshold 0; mBC: mean betweenness centrality; Q: modularity.

Measure	SW-stat	p-value
Cocaine consumption patterns		
Residualized Years.Begin	0.9865	0.7308
Residualized Coc.Age.Onset	0.9743	0.1881
Network metrics (Schaefer-232 parcellation)		
Residualized S_{neg}	0.9808	0.4428
Residualized Comp (thr = 0)	0.9819	0.4928
Residualized mBC	0.9821	0.5023
Residualized Q	0.9857	0.6872
Network metrics (Brainnetome-246 parcellation)		
Residualized S_{neg}	0.9926	0.9719
Residualized Comp (thr = 0)	0.9797	0.3322
Residualized mBC	0.9787	0.3539
Residualized Q	0.9898	0.8884

Supplementary Table 10. Hyperconnectivity test (CUD > HCT): Spearman's correlations between receptor density distributions (same as in the main text) and regional hyperconnectivity alterations in the integrated state using the Schaefer-232 atlas. P-values, derived from the spin test for cortical regions and random shuffling for subcortical regions, are FDR-corrected.

Source	Space	Neurotransmitter	Receptor	Corr	p-value
savli2012	MNI152	serotonin	5HT1a	0.173	0.3099
gallezot2010	MNI152	serotonin	5HT1b	0.213	0.0568
beliveau2017	fsaverage	serotonin	5HT2a	0.180	0.0568
beliveau2017	fsaverage	serotonin	5HT4	0.251	0.0361
radnakrishnan2018	MNI152	serotonin	5HT6	0.223	0.0260
beliveau2017	fsaverage	serotonin	5HTT	-0.065	0.7064
kantonen2020	MNI152	opioid	MOR	0.379	0.0025
ding2010	MNI152	norepinephrine	NET	-0.016	0.8889
gallezot2017	MNI152	histamine	H3	0.349	0.0025
smart2019	MNI152	glutamate	mGluR5	0.280	0.0057
dukart2018	MNI152	gaba	GABAa	-0.062	0.7064
norgaard2021	fsaverage	gaba	GABAa-bz	-0.028	0.8453
kaller2017	MNI152	dopamine	D1	0.124	0.5422
smith2017	MNI152	dopamine	D2	0.116	0.5678
sasaki2012	MNI152	dopamine	DAT	0.022	0.8216
normandin2015	MNI152	cannabinoid	CB1	0.351	0.0025
hillmer2016	MNI152	acetylcholine	A4B2	0.216	0.1021
naganawa2020	MNI152	acetylcholine	M1	0.238	0.0247
aghourian2017	MNI152	acetylcholine	VAChT	0.210	0.0361

Supplementary Table 11. Hypoconnectivity test (CUD < HCT): Spearman's correlations between receptor density distributions (same as in the main text) and regional hypoconnectivity alterations in

the integrated state using the Schaefer-232 atlas. P-values, derived from the spin test for cortical regions and random shuffling for subcortical regions, are FDR-corrected.

Source	Space	Neurotransmitter	Receptor	Corr	p-value
savli2012	MNI152	serotonin	5HT1a	-0.139	0.8582
gallezot2010	MNI152	serotonin	5HT1b	-0.180	0.5187
beliveau2017	fsaverage	serotonin	5HT2a	-0.102	0.9752
beliveau2017	fsaverage	serotonin	5HT4	0.083	0.9752
radnakrishnan2018	MNI152	serotonin	5HT6	0.055	0.9752
beliveau2017	fsaverage	serotonin	5HTT	0.025	0.9752
kantonen2020	MNI152	opioid	MOR	0.028	0.9752
ding2010	MNI152	norepinephrine	NET	-0.156	0.5216
gallezot2017	MNI152	histamine	H3	-0.030	0.9752
smart2019	MNI152	glutamate	mGluR5	0.005	0.9752
dukart2018	MNI152	gaba	GABAa	-0.058	0.9752
norgaard2021	fsaverage	gaba	GABAa-bz	-0.065	0.9752
kaller2017	MNI152	dopamine	D1	0.085	0.9752
smith2017	MNI152	dopamine	D2	-0.006	0.9752
sasaki2012	MNI152	dopamine	DAT	0.045	0.9752
normandin2015	MNI152	cannabinoid	CB1	-0.068	0.9752
hillmer2016	MNI152	acetylcholine	A4B2	-0.068	0.9752
naganawa2020	MNI152	acetylcholine	M1	0.060	0.9752
aghourian2017	MNI152	acetylcholine	VAChT	-0.037	0.9752

Supplementary Table 12. Hyperconnectivity test (CUD > HCT): Spearman's correlations between receptor density distributions (same as in the main text) and regional hyperconnectivity alterations in

the segregated state using the Schaefer-232 atlas. P-values, derived from the spin test for cortical regions and random shuffling for subcortical regions, are FDR-corrected.

Source	Space	Neurotransmitter	Receptor	Corr	p-value
savli2012	MNI152	serotonin	5HT1a	0.193	0.1539
gallezot2010	MNI152	serotonin	5HT1b	0.176	0.1423
beliveau2017	fsaverage	serotonin	5HT2a	0.109	0.4680
beliveau2017	fsaverage	serotonin	5HT4	0.297	0.0038
radnakrishnan2018	MNI152	serotonin	5HT6	0.186	0.0421
beliveau2017	fsaverage	serotonin	5HTT	0.060	0.6848
kantonen2020	MNI152	opioid	MOR	0.465	0.0010
ding2010	MNI152	norepinephrine	NET	-0.191	0.1091
gallezot2017	MNI152	histamine	H3	0.350	0.0019
smart2019	MNI152	glutamate	mGluR5	0.248	0.0142
dukart2018	MNI152	gaba	GABAa	-0.011	0.9261
norgaard2021	fsaverage	gaba	GABAa-bz	-0.052	0.6848
kaller2017	MNI152	dopamine	D1	0.308	0.0068
smith2017	MNI152	dopamine	D2	0.231	0.1451
sasaki2012	MNI152	dopamine	DAT	0.078	0.4807
normandin2015	MNI152	cannabinoid	CB1	0.392	0.0010
hillmer2016	MNI152	acetylcholine	A4B2	0.230	0.0715
naganawa2020	MNI152	acetylcholine	M1	0.141	0.1554
aghourian2017	MNI152	acetylcholine	VAChT	0.194	0.1554

Supplementary Table 13. Hypoconnectivity test (CUD < HCT): Spearman's correlations between receptor density distributions (same as in the main text) and regional hypoconnectivity alterations in

the segregated state using the Schaefer-232 atlas. P-values, derived from the spin test for cortical regions and random shuffling for subcortical regions, are FDR-corrected.

Source	Space	Neurotransmitter	Receptor	Corr	p-value
savli2012	MNI152	serotonin	5HT1a	-0.071	0.8626
gallezot2010	MNI152	serotonin	5HT1b	-0.195	0.2432
beliveau2017	fsaverage	serotonin	5HT2a	-0.113	0.7098
beliveau2017	fsaverage	serotonin	5HT4	0.005	0.9710
radnakrishnan2018	MNI152	serotonin	5HT6	-0.074	0.8005
beliveau2017	fsaverage	serotonin	5HTT	0.038	0.9387
kantonen2020	MNI152	opioid	MOR	0.148	0.5250
ding2010	MNI152	norepinephrine	NET	-0.211	0.2432
gallezot2017	MNI152	histamine	H3	0.077	0.8626
smart2019	MNI152	glutamate	mGluR5	-0.142	0.3568
dukart2018	MNI152	gaba	GABAa	-0.162	0.3568
norgaard2021	fsaverage	gaba	GABAa-bz	-0.172	0.3568
kaller2017	MNI152	dopamine	D1	0.046	0.9387
smith2017	MNI152	dopamine	D2	0.009	0.9710
sasaki2012	MNI152	dopamine	DAT	-0.086	0.7968
normandin2015	MNI152	cannabinoid	CB1	-0.003	0.9710
hillmer2016	MNI152	acetylcholine	A4B2	-0.064	0.8626
naganawa2020	MNI152	acetylcholine	M1	-0.039	0.9271
aghourian2017	MNI152	acetylcholine	VAChT	-0.012	0.9710

Reference:

1. Zamora-López, G., Chen, Y., Deco, G., Kringelbach, M. L. & Zhou, C. Functional complexity emerging from anatomical constraints in the brain: the significance of network modularity and rich-clubs. *Sci Rep* **6**, 38424 (2016).

2. Rubinov, M. & Sporns, O. Complex network measures of brain connectivity: Uses and interpretations. *NeuroImage* **52**, 1059–1069 (2010).

3. Girvan, M. & Newman, M. E. J. Community structure in social and biological networks. *Proceedings of the National Academy of Sciences* **99**, 7821–7826 (2002).
